# Mapping urban *Aedes aegypti* breeding containers: landscape-driven analysis in Bangkok, Thailand

**DOI:** 10.1186/s12942-026-00469-3

**Published:** 2026-05-13

**Authors:** Eric Daudé, Alexandre Cebeillac, Kanchana Nakhapakorn, Richard E. Paul

**Affiliations:** 1 Institut de recherche sur l’Asie du Sud-Est contemporaine, UMIFRE IRASEC, Centre National de la Recherche Scientifique (CNRS) - Ministère de l’Europe et des Affaires étrangères (MEAE), Bangkok, Thailand; 2 UMR IDEES 6266, Centre National de la Recherche Scientifique (CNRS) - Université de Rouen Normandie, Mont-Saint-Aignan, France; 3 Faculty of Environment and Resource Studies, Mahidol University, Bangkok, Thailand; 4 Ecology and Emergence of Arthropod-borne Pathogens unit, Institut Pasteur, Université Paris-Cité, Centre National de la Recherche Scientifique (CNRS), UMR 2000, Institut National de Recherche pour l’Agriculture, l’Alimentation et l’Environnement (INRAE) USC 1510, Paris, 75015 France

## Abstract

Urban dengue risk emerges from spatially heterogeneous interactions between mosquito vectors, the environment, and human activities. In Bangkok, *Aedes aegypti* breeding containers are predominantly artificial recipies whose distribution is shaped by the morphological structure of urban environments, including built-up density, compactness, interstitial open spaces, and vegetation configuration, as well as by everyday practices such as storage, waste accumulation, gardening, and maintenance. We tested whether a landscape-based typology of Bangkok defines functionally similar mosquito breedings containers by conducting standardized field surveys of outdoor potential breeding containers (PBC) across 36 urban zones (May 2023-December 2024). Among the 4,921 geolocated individual PBCs, small containers (< 1 L) dominated the container pool and accounted for most between-zone variability. PBC density showed a non-linear association with the built-up density and urban compactness gradient, with higher densities at both extremes of this Axis 1. We characterized container spatial aggregation using cluster metrics and classified zones into four aggregation profiles using PAM clustering. A multivariate model combining built intensity and PCA-based descriptors (|Axis 1| and Axis 3) explained a substantial fraction of the variability in PBC cluster coverage, enabling metropolitan-scale mapping of structurally favorable environments. Overall, urban morphology provides a meaningful baseline for identifying functional mosquito breeding containers, but local socio-environmental factors remain critical for explaining breeding-site aggregation.

**Supplementary Information:**

The online version contains supplementary material available at 10.1186/s12942-026-00469-3.

## Introduction

Dengue is a mosquito-borne viral disease caused by four closely related dengue virus serotypes and transmitted primarily by *Aedes aegypti* in urban environments. In Thailand, dengue remains endemic and is characterized by recurrent seasonal outbreaks, particularly during and after the rainy season, when climatic conditions and water availability favor vector development [11]. The transmission cycle depends on repeated interactions between infected humans and anthropophilic mosquitoes, whose life cycle is closely associated with artificial water-holding containers commonly found in and around dwellings. As a result, urban environmental conditions play a central role in shaping local transmission potential.

In urban settings, dengue transmission is not simply the outcome of vector abundance or human density, but rather the product of a complex and spatially structured pathogenic system [9]. Viral circulation emerges from the intermittent alignment of multiple processes operating at different scales: mosquito population dynamics [39], human exposure and mobility [38], immunity shaped by the co-circulation of several serotypes, and micro-environmental conditions that modulate vector survival [5, 13]. As a consequence, stable and monotonic relationships between entomological indicators and dengue incidence are rarely observed, even in endemic settings [33].

Urban areas constitute the primary arenas of dengue transmission, as *Aedes* mosquitoes are highly adapted to anthropized environments [3]. Rather than occupying urban space homogeneously, they exploit a mosaic of micro-environments structured by the morphological structure of the built environment, including building density, compactness, and the organization of interstitial open spaces [23, 25] and everyday human practices [1]. Breeding containers are predominantly artificial, produced unintentionally by domestic water storage, waste accumulation, construction activities, and the maintenance - or neglect - of interstitial open spaces between and around buildings [14, 31, 37]. Consequently, the distribution and density of these potential breeding containers (PBC) are unlikely to be purely random, and their enumeration provides an operational proxy for estimating local vector abundance [12] and, by extension, potential transmission risk, forming the empirical basis for the construction of standard entomological indicators [19].

A central difficulty in dengue research lies not in the absence of indicators, but in the spatial frameworks within which these indicators are constructed and interpreted [17]. Entomological indices such as the Container Index (percentage of water-holding containers positive for immature stages), the House Index (percentage of houses with at least one positive container), or the Breteau Index (number of positive containers per 100 inspected houses) are classical tools in dengue surveillance. However, they are also aggregation products: they transform fine-scale entomological observations into summary measures computed over spatial units that are typically defined by technical, operational, or administrative constraints (e.g. Bowman et al., [6] rather than by ecologically meaningful boundaries. As a result, the spatial signal they capture may be shaped as much by the size, shape, and boundaries of the reporting units used for aggregation, whether defined as households, neighborhoods, surveillance sectors, or administrative districts, as by the underlying ecology of vector habitats.

The choice of an appropriate spatial unit to relate dengue cases to associated risk factors, most notably larval indices, therefore has a strong influence on both the outcomes and the interpretability of statistical analyses [30]. Empirical studies examining the relationship between variations in the Breteau Index and dengue incidence report highly inconsistent results across spatial scales. At the household level, this relationship has been found to be either positive [7, 26] or negative [20, 29]. At the neighborhood level, studies likewise report both positive [32] and negative associations [2]. At broader spatial scales - districts, administrative units, or entire cities - the relationship often becomes weak or non-significant [8, 21, 34].

These scale-dependent and sometimes contradictory findings strongly suggest that the statistical relationship between larval indices and dengue incidence is highly sensitive to the choice of spatial unit, reflecting aggregation effects rather than intrinsic epidemiological mechanisms [16]. The choice of spatial unit profoundly affects both the magnitude and the direction of observed relationships between vectors, environment, and disease, a phenomenon encapsulated in the Modifiable Areal Unit Problem [24]. When heterogeneous urban environments are aggregated into a single spatial unit, local processes governing vector persistence and human exposure are diluted, and the resulting indicators lose much of their explanatory power.

This observation calls for a shift in perspective, in which spatial units are defined a priori from vector ecology rather than imposed administrative boundaries, and urban space is conceptualized as a mosaic of functional habitats with contrasted mosquito carrying capacities. By defining habitats from the resources required to complete the mosquito life cycle, the Resource-Based Habitat Concept [15, 28] reframes urban space from the vector’s perspective, placing potential breeding containers at the core of vector carrying capacity as emergent products of urban morphology and human activity. In this study, urban morphology refers to the spatial configuration of the urban environment, including the density and arrangement of buildings, the distribution of open and vegetated spaces, and the organization of interstitial micro-spaces likely to condition the presence and aggregation of potential breeding containers.

**Fig. 1:**
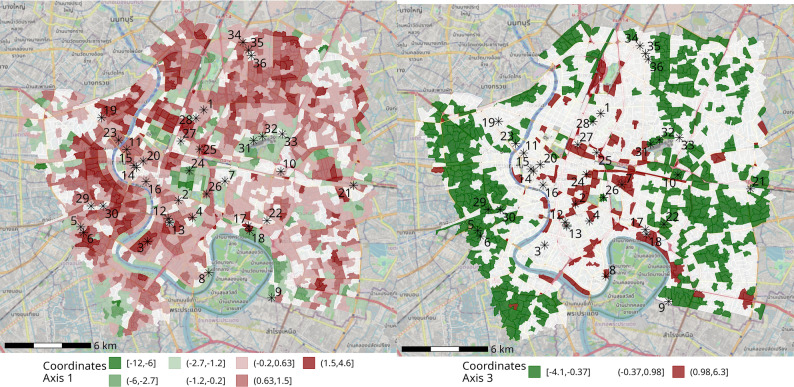
Spatial distribution of factorial coordinates for Axis 1 (built-up density and urban compactness gradient) and Axis 3 (internal configuration of built and vegetated spaces) across the Bangkok metropolitan area. Axis 1 highlights the large-scale center-periphery structure associated with variations in building density and inter-building space, while Axis 3 reveals finer-scale differences in the spatial organization and fragmentation of vegetated and interstitial areas within urban morphology (from [10]). The spatial units correspond to morphologically homogeneous zones (~ 19.6 ha; ~250 m radius), corresponding to a local spatial scale relevant for mosquito habitat processes and consistent with the typical dispersal range of Aedes aegypti. The numbers represent the ID of the surveyed zones

Building on this conceptual foundation, we previously developed a typology of the urban landscape of Bangkok designed to reflect functionally contrasted mosquito habitats [10, 22]. Here, typology refers to a classification of urban spatial units into groups sharing similar morphological and spatial characteristics, derived from quantitative descriptors of the built environment and vegetation structure. In that previous work, the city was partitioned into spatial units coherent with mosquito dispersal constraints and further subdivided into homogeneous zones based on built density, building height, interstitial open spaces, and vegetation structure (Fig. 1). This work revealed that Bangkok’s urban morphology is structured along several complementary gradients [10]. Principal Component Analysis (PCA) was used as a dimensionality reduction method to synthesize multiple correlated variables describing urban morphology into a smaller set of independent axes. Each axis represents a dominant gradient of variation in the urban landscape, combining information on building density, vegetation structure, and spatial configuration. This approach allows complex urban environments to be described through a limited number of interpretable dimensions, which were subsequently used to construct the typology and guide both the sampling design and the statistical analyses. Axis 1 of the principal component analysis captures a broad center-periphery pattern associated with built-up density and urban compactness, distinguishing highly consolidated central fabrics from more open peripheral environments. Axis 2 primarily reflects the amount and fragmentation of vegetation cover, independently of built-form variables. It distinguishes areas with relatively dense and continuous vegetation from those where vegetation is sparse or highly fragmented. Axis 3, by contrast, captures the internal spatial configuration of built and vegetated spaces within the urban fabric. It differentiates environments where vegetation is organized into larger, more continuous patches from those where it is distributed as small, spatially scattered green elements embedded within built-up areas, such as small gardens, roadside vegetation, isolated planted spaces, or fragmented vegetated margins. Because our survey focuses on anthropogenic environments where artificial containers are produced, maintained, and accumulated, we prioritized the axes that most directly capture the organization of built-up space and its associated interstitial environments. In this perspective, Axis 1 and Axis 3 were considered more relevant than Axis 2 for structuring the PBC sampling design.

This typology does not aim to describe the city in conventional morphological or socio-economic terms, but to identify urban environments that are functionally similar from the perspective of mosquito ecology [35]. Zones sharing comparable landscape configurations are hypothesized to generate similar potentials for the production of breeding containers, even when geographically distant, whereas adjacent zones with contrasting configurations may exhibit markedly different distribution of breeding containers. Within this framework, socio-demographic factors are not ignored but are assumed to modulate these patterns rather than fully determine them when urban landscape configurations are comparable.

In the present study, we use this typology as a sampling and analytical framework to test whether these spatial units correspond to differentiated patterns of potential breeding container abundance and spatial organization. Building on this typology, we then designed a field survey to empirically examine how outdoor potential breeding containers (PBC) are distributed across the contrasted urban configurations identified through spatial analysis. Specifically, we sought to determine whether gradients in building density and vegetation structure translate into distinct and internally consistent profiles of breeding-site abundance, diversity, and spatial organization observed on the ground. By combining high-resolution landscape characterization with standardized surveys of outdoor oviposition containers within a stratified sample of urban zones, the study tests whether functional urban habitats correspond to coherent entomological signatures, independently of their geographic proximity. In doing so, we move from a conceptual classification of urban environments to its empirical validation and operationalization, providing the basis for the methodological approach detailed in the following section.

## Methods and field data collection

The aim of the field study was to empirically assess whether urban environments characterized by contrasted building density and vegetation configuration effectively discriminate the diversity, density, and spatial organization of outdoor potential breeding containers (PBC).

### Study area and sampling design

**Fig. 2:**
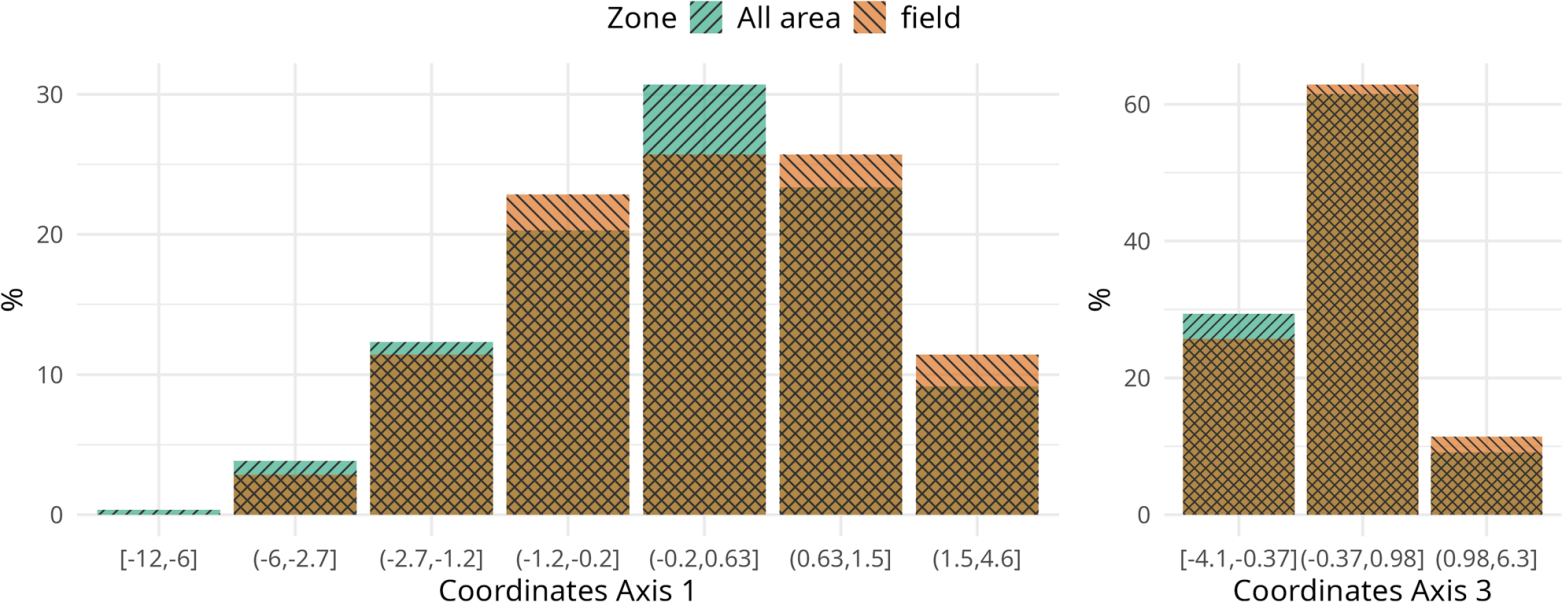
Comparison between the full urban landscape typology and the field-sampled zones along the main typological gradients. Relative distribution (%) of all urban zones (green) and field-surveyed zones (orange) across classes of Axis 1 (left; gradient of built-up density and urban compactness, from open fabrics with large inter-building distances to highly consolidated urban cores) and Axis 3 (right; gradient of internal vegetation configuration, from fragmented and dispersed green elements to larger, more continuous vegetated patches within urban fabrics)

A total of 36 urban zones (Fig. 1) were selected for a pilot field survey conducted between May 2023 and December 2024 (see Appendix 1, Figure S1 which illustrates the coverage and representativeness of the sampling design). The sampling strategy was designed to ensure that the proportion of selected zones within each class of the typology closely reflected their distribution across the entire study area. In this way, the field sample preserves the structural balance of the urban landscape as captured by the factorial analysis (Fig. 2).

**Fig. 3:**
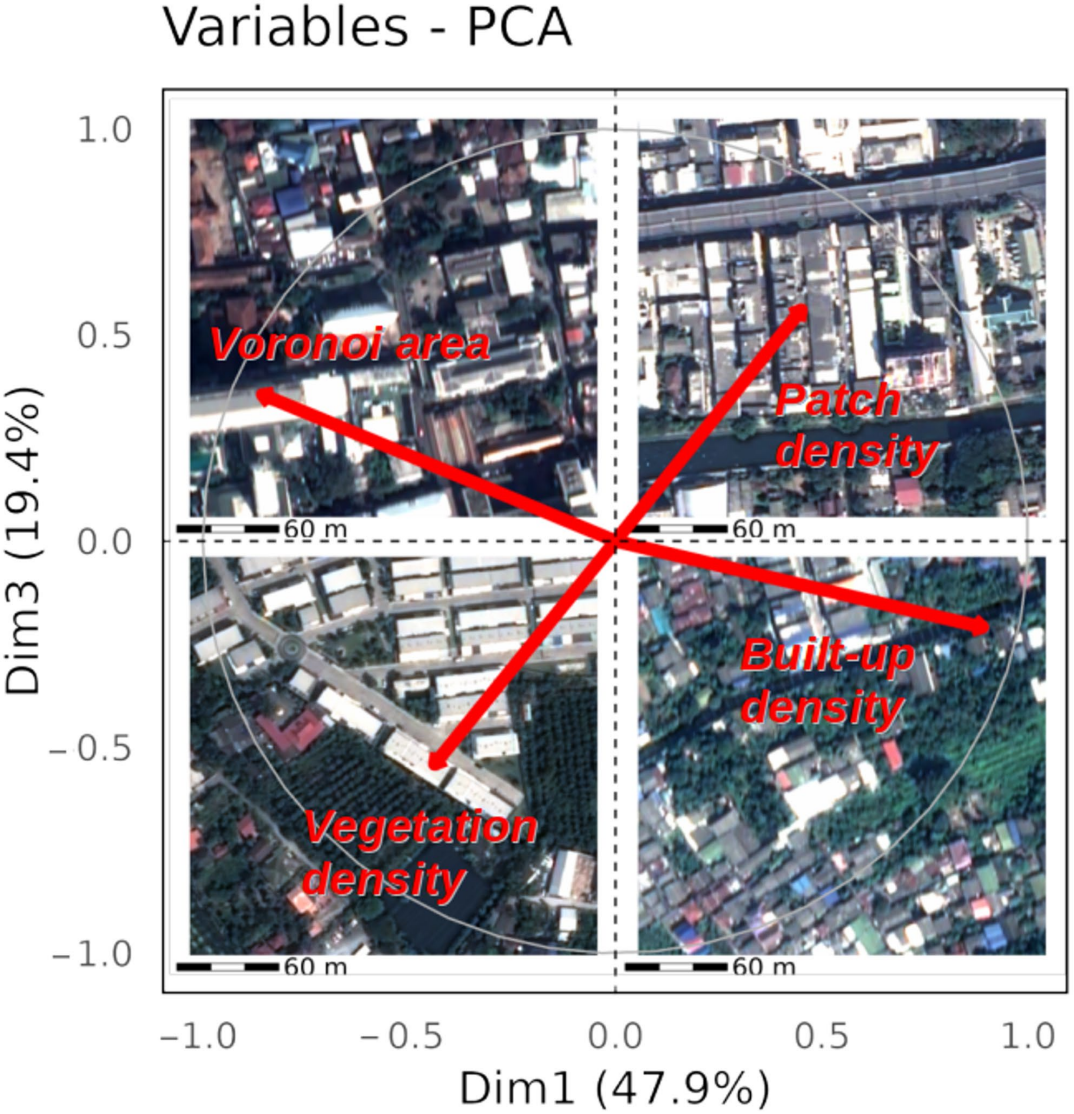
Principal Component Analysis (PCA) of building- and vegetation-related variables structuring urban landscapes in Bangkok. PCA summarizes multiple correlated variables into a reduced set of independent axes representing dominant spatial gradients. The first axes represent the main gradients of variation in urban morphology, combining information on built density, vegetation structure, and spatial configuration

The selected zones collectively form a transect along the first principal axis of the typology, which represents the dominant gradient of built-up density and urban compactness, contrasting areas with large inter-building distances and abundant open space with highly consolidated fabrics. At the same time, the selection explicitly incorporated variation along the third axis, which differentiates urban environments according to the internal configuration of vegetation, ranging from continuous large vegetated patches to dispersed and fragmented green elements (Fig. 3).

By combining proportional representation with coverage of both axes, the sampling design ensures that the pilot survey captures the diversity of morphological configurations present in the metropolitan area, without over or under-representing any segment of the urban gradient.

Practical constraints related to fieldwork logistics were also taken into account. To ensure feasibility within the survey period, selected zones were required to be located within approximately one hour of travel time from the city center using public transportation (metro and BTS) or taxis. In addition, the accessibility and internal configuration of each zone were systematically examined prior to field deployment using Google Street View. To ensure feasibility and consistency of field surveys, data collection was restricted to publicly accessible neighborhoods, allowing the application of standardized protocols across sites while covering a wide range of urban landscape configurations.

### Field survey protocol

Field surveys were designed to assess whether urban environments, characterized by building density and vegetation configuration, effectively differentiate the abundance, volume capacity, and spatial distribution of outdoor PBC. These were defined as any artificial container capable of holding water and thus suitable for *Aedes* oviposition, regardless of whether water originated from rainfall or human-related activities.

In Bangkok, common outdoor breeding containers include discarded tyres, flower pot saucers, jars, buckets, skips, and a wide range of small waste items such as cups, coconuts, or food trays. Given this diversity, we adopted a functional approach based on container volume capacity rather than container type. Our analysis focuses on (i) the number of containers, (ii) their volume capacity, and (iii) their spatial distribution within each urban zone. These attributes jointly influence both the likelihood of oviposition and the overall water availability for larval and pupal development, approximated through the distribution of containers across predefined volume classes.

Accordingly, each observed PBC was geolocated and assigned to a predefined volume category (Table 1) based on visual estimation, without direct measurement.

**Table 1:**
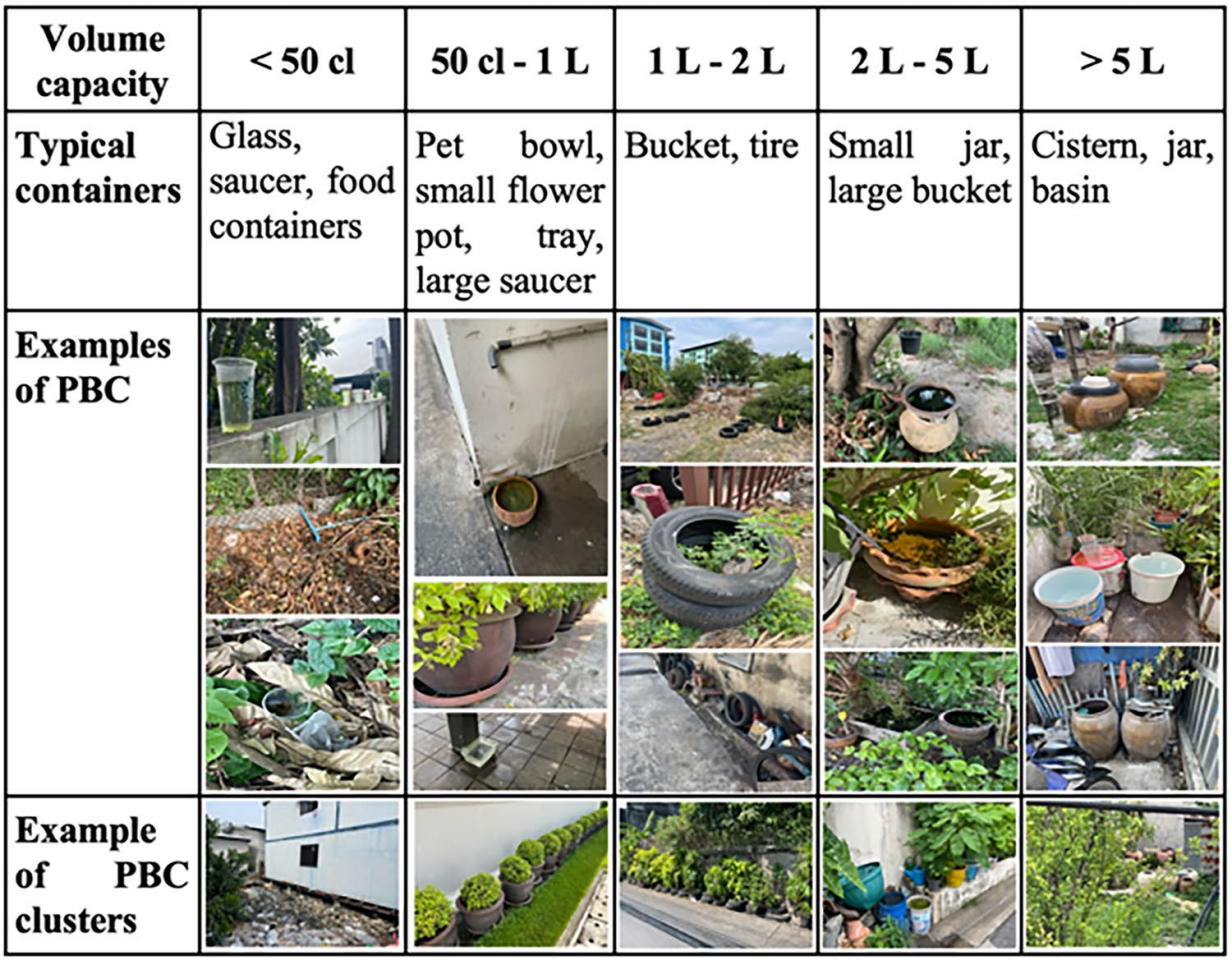
Typology of outdoor potential breeding containers and associated volumetric capacity classes (photos: É. Daudé, 2024, Bangkok)

For each selected urban zone, surveyors walked between 2 and 4 km, depending on neighborhood size and accessibility, with the objective of identifying as many outdoor PBC as possible. Survey effort was standardized by expressing PBC abundance as the number of containers per volume category (Table 1) per kilometer walked. GPS tracks were continuously recorded and checked against zone boundaries to ensure spatial consistency.

All PBCs were geolocated using a standardized protocol implemented through QField, allowing the simultaneous recording of survey routes and individual container positions. Observations were restricted to containers visible from surveyed routes and adjacent accessible spaces (e.g., roadside edges, courtyards, gardens, interstitial areas). Detection therefore depended on local visibility and accessibility conditions, which varied according to urban configuration (e.g., walls, fences, vegetation cover).

Only outdoor artificial containers were recorded, natural oviposition sites such as plants or tree holes were excluded.

This protocol enabled the quantification, for each surveyed zone, of (i) the relative contribution of each volume category and (ii) the standardized density of PBC per kilometer.

Inter-observer consistency was assessed on a subset of surveyed zones (*n* = 16) through independent surveys conducted along identical routes. The results showed strong agreement in both container counts and composition (r^2^ = 0.88), indicating good reproducibility.

### Data acquisition tools and outputs

Data acquisition was standardized using QField, enabling the simultaneous recording of survey routes and the geographic position of individual PBC in the field. This protocol provided the spatially explicit data required for subsequent analyses, including container density, spatial distribution, clustering, and surface coverage.

For each surveyed zone, the final dataset includes: (i) the survey route, (ii) the total distance covered, (iii) the number of outdoor PBC per volume category standardized per kilometer, and (iv) the geographic coordinates of individual containers. Appendix 1 (Figure S2) summarizes the spatial coverage of the survey, including routes, sampling effort, and the distribution of PBC across volume categories.

### Spatial clustering of PBC

**Fig. 4:**
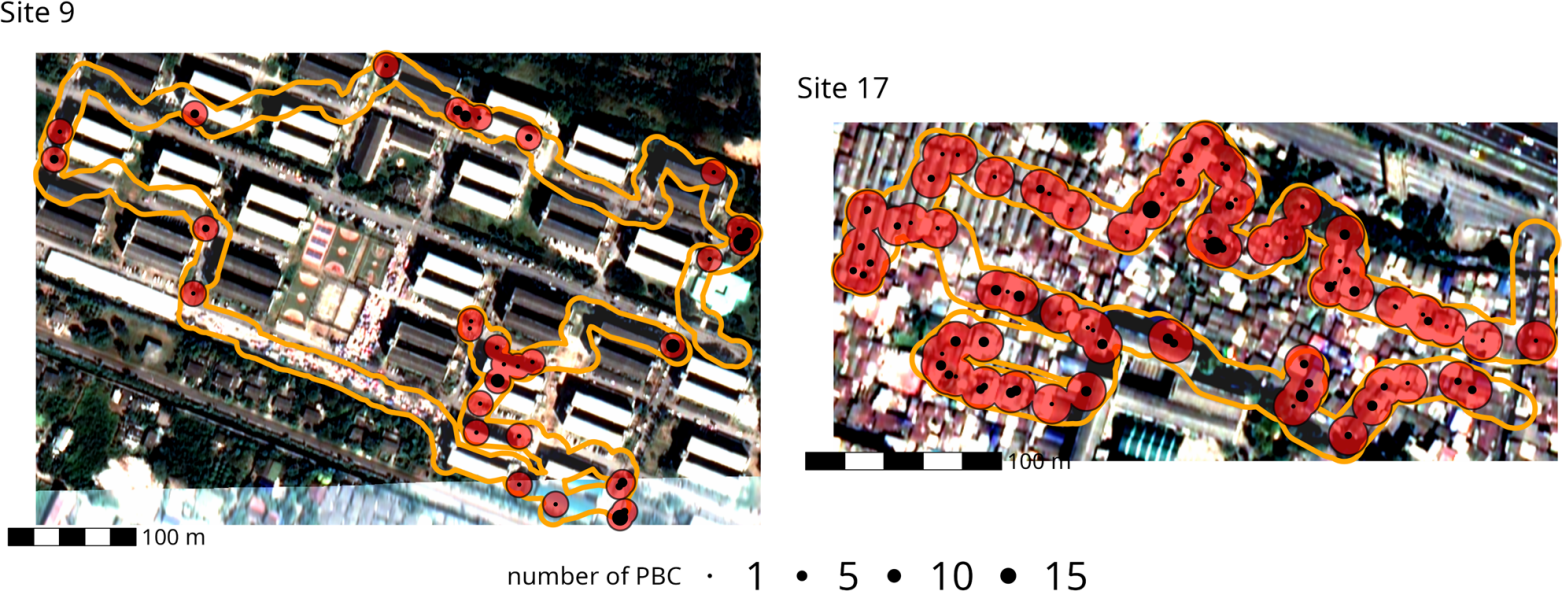
Schematic illustration of PBC clustering based on a 10-m buffer approach. Examples from two surveyed zones (left, Zone 9 and right, Zone 17) showing the surveyed routes (orange outlines) and geolocated PBC (black dots). A 10-m buffer was applied around each PBC (red areas), and intersecting buffers were merged to define clusters of potential breeding containers. This approach captures contrasts between dispersed configurations (Zone 9) and highly clustered distributions along streets (Zone 17)

Buildings act as elementary units of PBC production and may generate few or many containers in their immediate surroundings. At the neighborhood scale, this can result either in localized concentrations of PBC or in their dispersion along linear urban features such as streets (Fig. 4).

To characterize the spatial distribution of PBC, clusters were constructed by applying a 10-m buffer around each geolocated PBC and merging intersecting buffers. This approach allows the identification of spatially aggregated breeding containers. For each surveyed zone, we derived the number of PBC clusters per kilometer of surveyed route, the number of PBC per cluster, and the cluster surface area. PBC cluster coverage was defined as the proportion of the buffered surveyed-route area intersected by merged PBC cluster polygons. A relative cluster size was also computed by normalizing cluster area by the buffered surface of the surveyed route, enabling comparison between zones with different route lengths.

### Statistical analysis of PBC density and landscape gradients

To examine how PBC abundance varied across the main urban landscape gradients, we analyzed the relationship between the number of PBC per kilometer and the PCA-derived landscape axes used in the sampling design, particularly Axis 1 and Axis 3. These two axes were selected because they capture, respectively, the center-periphery gradient of urban compactness and the internal spatial configuration of built and vegetated spaces. Exploratory regression analyses included both linear and second-degree polynomial models to assess potential non-linear relationships between PBC density and PCA-derived landscape gradients.

### Classification of PBC spatial configurations (PAM)

To identify recurrent types of PBC spatial organization across surveyed zones, we applied a Partitioning Around Medoids (PAM) clustering procedure [18] to a set of variables describing the structure of PBC clusters (e.g., number of clusters per kilometer, mean number of PBC per cluster, cluster size, and cluster coverage). PAM was preferred because it is robust to outliers and suitable for identifying representative groups in relatively small datasets [27]. The resulting classes were used to summarize contrasted spatial configurations of breeding container distribution across the sampled urban environments.

### Modeling and extrapolation of PBC cluster coverage

To estimate the spatial coverage of PBC clusters beyond surveyed routes, we fitted a linear regression model relating the observed proportion of surveyed-route area covered by PBC clusters to three explanatory variables calculated along each surveyed route: built-up linear density, the absolute value of PCA Axis 1, and PCA Axis 3.

The resulting relationship was first calibrated on the 36 surveyed routes. It was then extrapolated to the full set of urban zones by replacing route-based explanatory values with the corresponding values calculated at the zone level. This allowed us to estimate the expected proportion of space potentially covered by PBC clusters across Bangkok.

To assess the coherence of this extrapolation across spatial scales, we compared the zone-level predicted values with the observed route-level values for the 36 sampled zones. This comparison was intended as a consistency check of scale transfer, rather than as an independent predictive validation.

## Results

This section describes the abundance, volume capacity, and spatial distribution of outdoor potential breeding containers across the surveyed urban zones.

### Abundance and volume structure of potential breeding containers

A total of 4,921 PBC were recorded and geolocated across the 36 zones. Their distribution by volume class reveals two complementary patterns: a strong dominance of small containers in absolute abundance, and substantial inter-site variability in the relative composition of volume classes.

**Fig. 5:**
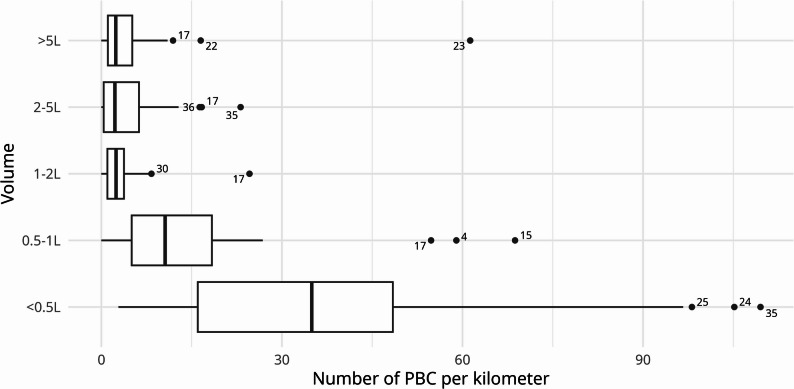
Distribution of outdoor potential breeding containers (PBC) by volume category and density per km. Boxplots show the number of PBC per kilometer walked for each volume capacity class, across all surveyed urban zones. Points represent outliers

Across the 36 surveyed urban zones, the average number of outdoor PBC per km walked varied according to volume capacity (Fig. 5). Mean densities range from approximately 1 container per km for the largest class (> 5 L) to around 19 containers per km for the smallest class (< 0.5 L). Small-capacity PBCs (less than 1 L) were systematically the most abundant across all zones and constitute the primary driver of variability between sites.

The density of small containers (< 1 L) showed pronounced heterogeneity, with values ranging from 3 to approximately 110 PBC per kilometer. This size class therefore contributes most strongly to the differentiation of zones in terms of overall breeding-site density. Several zones exceeded the ninth decile of the distribution, indicating locally high concentrations of small-capacity containers. This was particularly the case for zone 35 (Chan Kasem) characterized by small detached houses where numerous flowerpots were observed along the streets, substantially increasing the presence of small potential breeding containers.

**Fig. 6:**
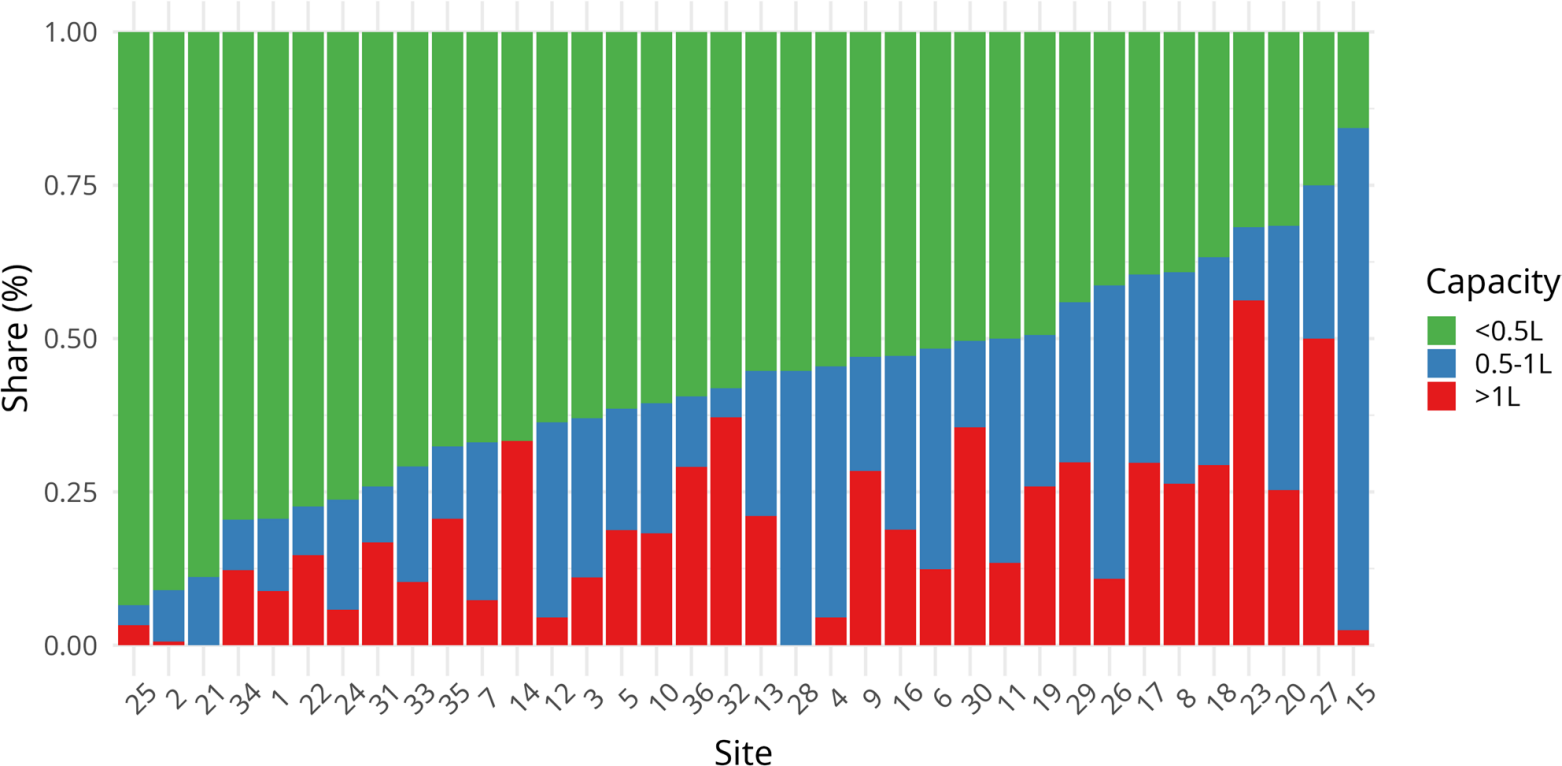
Relative proportion of outdoor potential breeding containers by volume capacity across surveyed sites. Stacked bars show, for each surveyed site, the proportion of potential breeding containers belonging to three volume classes (< 0.5 L, 0.5–1 L, and > 1 L). Sites are ordered by decreasing proportion of small containers

When considering all zones together, containers with a volume capacity greater than 1 L represent less than one-sixth of all recorded PBC (Fig. 6). However, their relative contribution varies substantially across sites. Zones 23 (Wat Daowaduengsaram, 121 PBC/km) and 27 (Victory Monument-Ramathibodi, 11 PBC/km) are notable in this regard: in both zones, approximately 50% of recorded PBC belonged to the > 1 L category. This similarity in proportional structure contrasts sharply with the large difference in absolute abundance between the two sites.

Taken together, these findings highlight strong between-zone contrasts not only in the density of potential breeding containers, but also in their volume structure. Urban zones differ markedly in the relative contribution of small, medium, and large containers, resulting in distinct local profiles of potential water availability, which may in turn influence vector productivity.

### Relationship between the urban landscape gradient and potential breeding container density

**Fig. 7:**
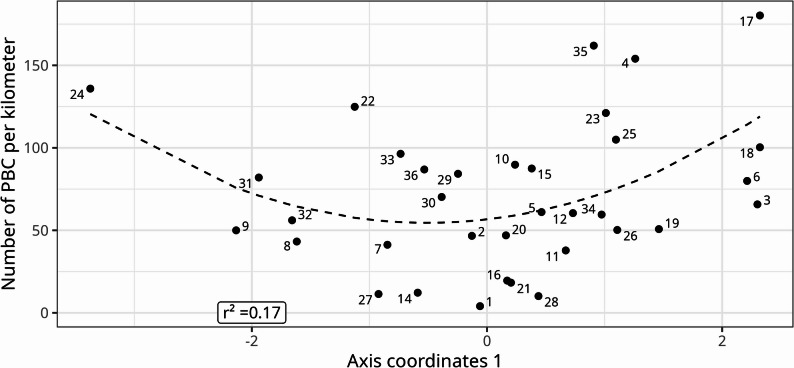
Relationship between the urban landscape gradient (PCA Axis 1, representing the built-up density and urban compactness gradient) and potential breeding container density. The dashed line represents the result of the quadratic regression, suggesting higher PBC densities toward both extremes of Axis 1

The number of outdoor potential breeding containers per kilometer shows a contrasted relationship with PCA Axis 1, which represents the main gradient of built-up density and urban compactness across the study area (Fig. 7). When modelling the relationship between the two factors using a second-degree polynomial, a non-linear pattern emerges (R² = 0.19). The fitted curve suggests a U-shaped relationship, with higher PBC densities observed at both extremes of the compactness gradient and lower values around intermediate axis coordinates. Although the explanatory power of Axis 1 alone remains moderate, this quadratic specification shows that the effect of urban compactness on PBC density is not monotonic. Instead, aggregation processes appear to intensify in morphologically extreme contexts. The moderate dispersion of points around the fitted curve nevertheless indicates that additional structural or local factors contribute to the observed variability, justifying the inclusion of further morphological descriptors in subsequent models.

In contrast, no significant relationship was observed between PBC density and Axis 3 (R² = 0.012), indicating that this gradient does not directly structure overall container abundance. This result confirms that this axis does not directly structure overall PBC density, but rather captures differences in the internal spatial organization of urban environments, which become relevant when considering the aggregation and spatial distribution of containers.

### Spatial coverage and linear density of potential breeding containers along the urban landscape gradient

**Fig. 8:**
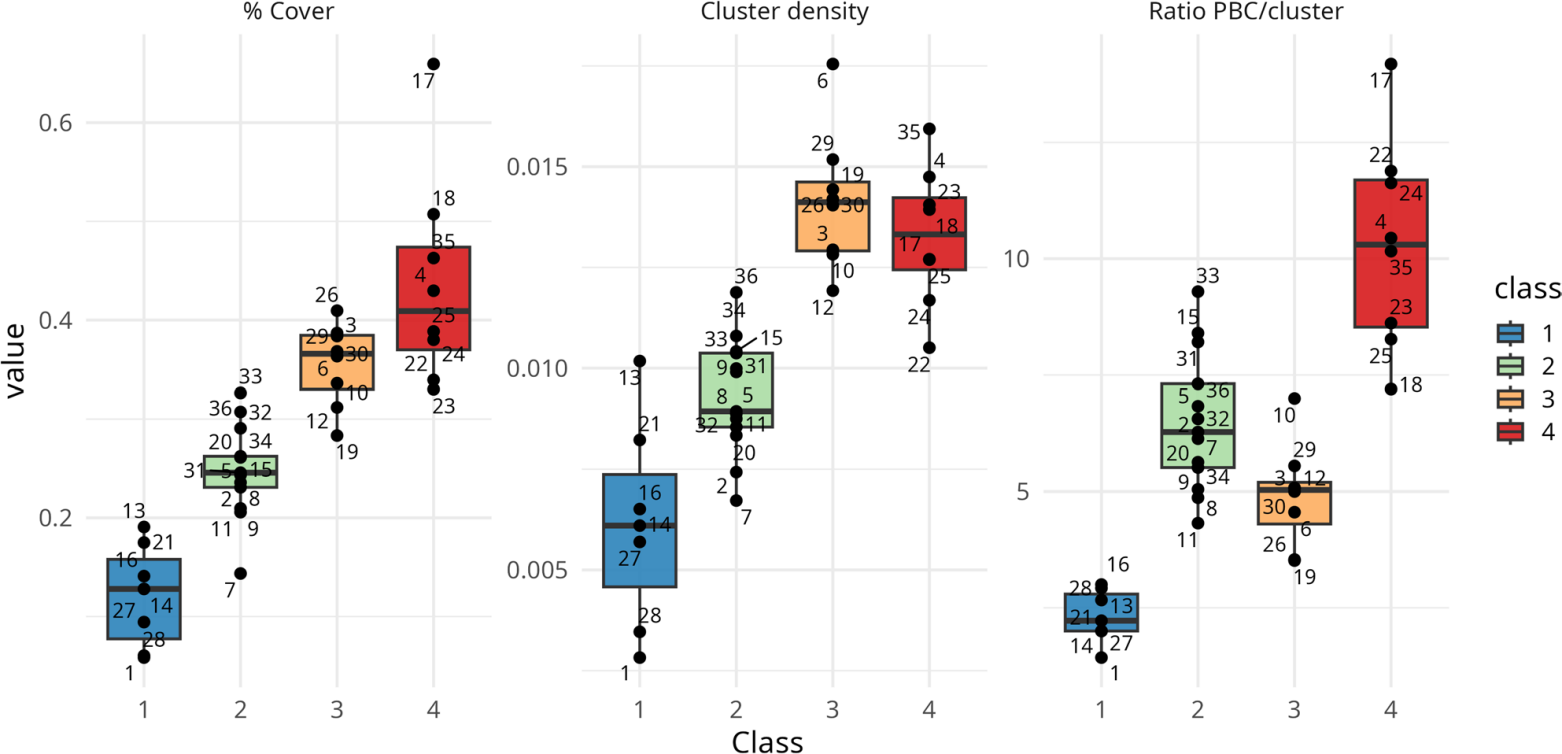
PAM-based classification of surveyed urban zones according to spatial aggregation patterns of potential breeding containers. Boxplots display the distribution of three complementary indicators across the four classes identified by the PAM algorithm: percentage of buffered surveyed-route area covered by PBC clusters; cluster density; and mean number of PBC per cluster. Points correspond to individual surveyed zones (site IDs)

Sites were classified using three complementary indicators. First, the percentage of buffered surveyed-route area covered by PBC clusters (% Cover) captures the spatial footprint of aggregated breeding containers and reflects the extent to which PBC are spatially concentrated along the surveyed route environment. Second, the cluster density describes the frequency of aggregation structures across the zone, independently of their size. Finally, the PBC per cluster ratio measures cluster intensity, i.e. the average number of containers within each cluster, providing information on local accumulation (Fig. 8).

Together, these three parameters characterize distinct dimensions of PBC spatial organization: extent (how much of the buffered surveyed-route is covered by clusters), frequency (how many clusters per unit area), and intensity (how many PBC per cluster). The PAM procedure then groups sites that share similar configurations across these dimensions, allowing the identification of four structurally distinct cluster profiles.

Sites in Class 1 exhibit the lowest values across all three indicators and correspond to zones where PBC are sparse, weakly aggregated and spatially limited. Both the extent and intensity of clustering are minimal. Sites in class 2 are characterized by more frequent aggregation structures, but clusters remain relatively small in size. The spatial footprint increases, yet intensity remains moderate. Class 3 configuration reflects environments where clusters are numerous and spatially extended, but each cluster contains a moderate number of containers. It suggests a fine-grained, spatially repetitive aggregation process compared to the clearly distinct class 4 for which the sites combine large spatial footprint with high internal cluster intensity. They represent environments where PBC are not only frequent but also strongly concentrated within clusters. From an operational perspective, this typology is particularly informative, as it distinguishes environments where control strategies might need to target either numerous small clusters (class 3) or fewer but highly populated clusters (class 4).

**Fig. 9:**
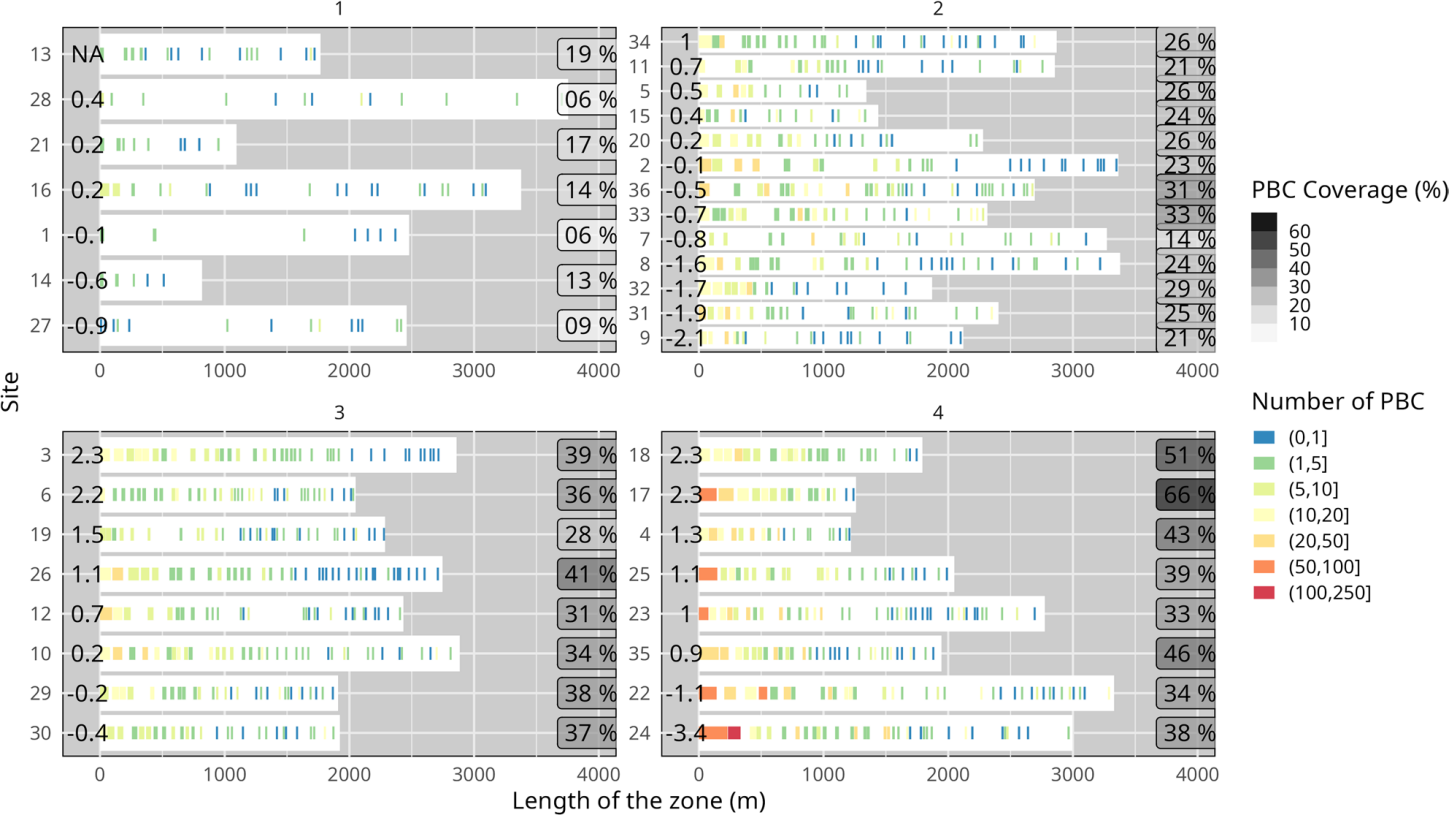
Spatial distribution and intensity of PBC clusters along surveyed routes. Each panel corresponds to one of the four PAM-derived aggregation classes (1 to 4). Within each panel, horizontal bars represent individual surveyed zones (site IDs indicated on the left). The x-axis shows the cumulative length of the surveyed route within each zone (in meters). Colored vertical ticks indicate the position and intensity of PBC clusters along the route, producing a linear representation of container distribution along accessible paths. This visualization highlights contrasts between dispersed configurations and highly clustered aligments, typically associated with linear urban features such as streets or parcel boundaries. Colors correspond to the number of PBC per cluster, categorized into seven classes, with white indicating PBC-free segments

The spatial distribution of PBC along the surveyed routes reveals the spatial counterpart of the PAM classification for each class (Fig. 9). This representation can be interpreted as a linear spatial profile of PBC distribution along surveyed routes, allowing comparison of clustering patterns between zones. Each horizontal bar represents a zone, with colored ticks indicating cluster intensity, and the grey box percentage representing overall PBC coverage. Class 1 has a low aggregation profile, with a sparse and discontinuous cluster signal along routes. Colored marks are scattered and mostly in low-density categories (blue/green), while coverage percentages remain low (< 20%). Class 2 exhibits more frequent clusters distributed along larger portions of the routes. Coverage increases (approx. 20–30%), but intensity remains moderate. Clusters are present throughout the zone without forming highly concentrated hotspots. Class 3 displays a dense and relatively continuous spatial signal. Numerous clusters are distributed across most of the route length, and coverage values are high (approx. 30–40%). However, extreme intensity clusters remain limited, indicating frequent but moderately sized aggregation nodes. Finally, class 4 is clearly distinct with routes showing extended segments with dense and often high-intensity clusters (including orange/red categories). Coverage values are the highest (up to 66%), confirming that these zones combine strong spatial extent with high internal cluster intensity.

Overall, these results show that urban zones differ not only in the total number of PBCs, but also in the way these containers are spatially arranged along accessible paths. The coexistence of zones with comparable linear coverage but contrasting spatial configurations - some characterized by dispersed containers, others by highly aggregated clusters - underscores the importance of jointly considering coverage, density, and intra-zone spatial clustering when characterizing breeding-site availability in urban environments.

### Estimating PBC cluster coverage across Bangkok

**Fig. 10:**
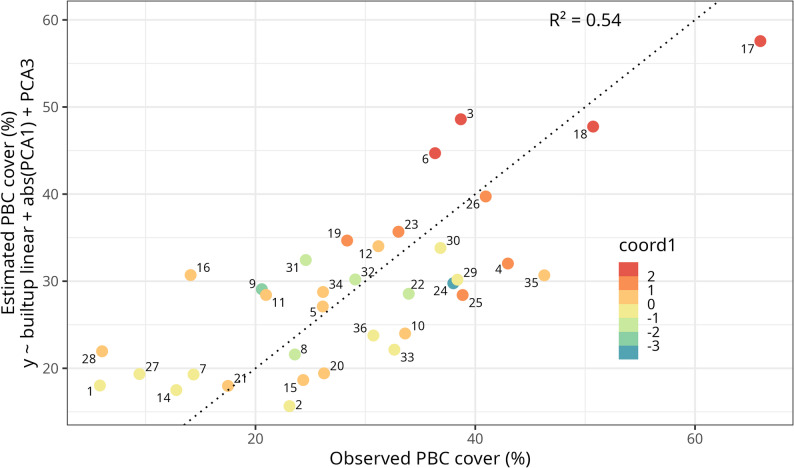
Relationship between observed and fitted PBC cluster coverage across the 36 surveyed routes. Scatterplot comparing observed PBC coverage (%) (x-axis) with fitted coverage (%) derived from a linear model including built-up density and PCA-based descriptors (y-axis). Each point represents one surveyed zone (ID site). Colors correspond to the coordinate value on the first principal axis (coord1), reflecting the built-up density gradient from open (blue/green) to highly compact (orange/red). The dotted line represents the 1:1 agreement line

To synthesize these results, we first examined the relationship between the surface covered by PBC clusters and the linear density of buildings across surveyed zones (i.e. the number of buildings from Google Open Buildings^1^ within a 10 m radius from the traveled path, divided by the length of the traveled path). Building density alone explains a moderate but meaningful fraction of the variability in PBC coverage (R² = 0.41), confirming a positive association between building density and the spatial extent of potential breeding containers. However, when incorporating additional structural descriptors derived from the principal component analysis, i.e. the absolute values of Axis 1 capturing the intensity of the compactness gradient and the coordinates on Axis 3 reflecting internal configuration of built and vegetated spaces, the explanatory power of the model increases to R² = 0.54 (Fig. 10). This improvement indicates that PBC cluster coverage is not solely driven by building density per se, but also by the broader morphological configuration of urban space, while the internal configuration of built and vegetated spaces, captured by Axis 3, makes a weaker and only marginally significant contribution to the model (Table 2).

The linear regression model was first calibrated on the 36 surveyed routes, using route-level PBC cluster coverage as the response variable and route-level values of built-up linear density, |Axis 1|, and Axis 3 as predictors (Table 2). The fitted relationship was then used as the basis for spatial extrapolation beyond the sampled routes.

**Table 2:**
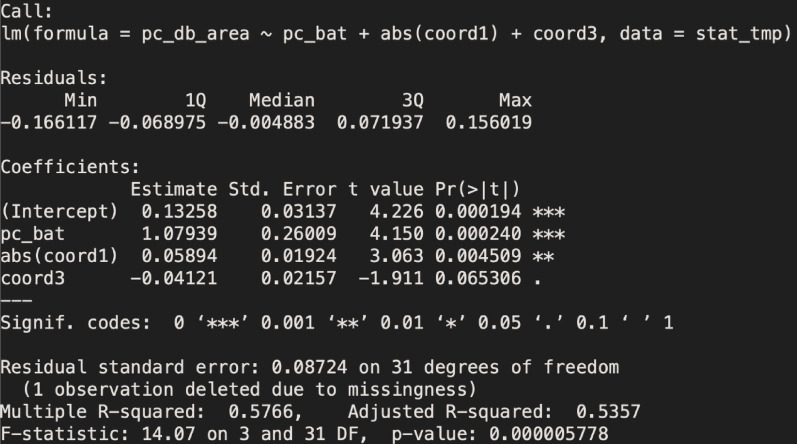
Linear regression model explaining PBC cluster coverage. Linear model relating PBC cluster coverage to built-up linear density (pc_bat) and PCA-derived descriptors of urban morphology (|coord1| and coord3). Coefficients, standard errors, significance levels, and model fit statistics are reported

**Fig. 11:**
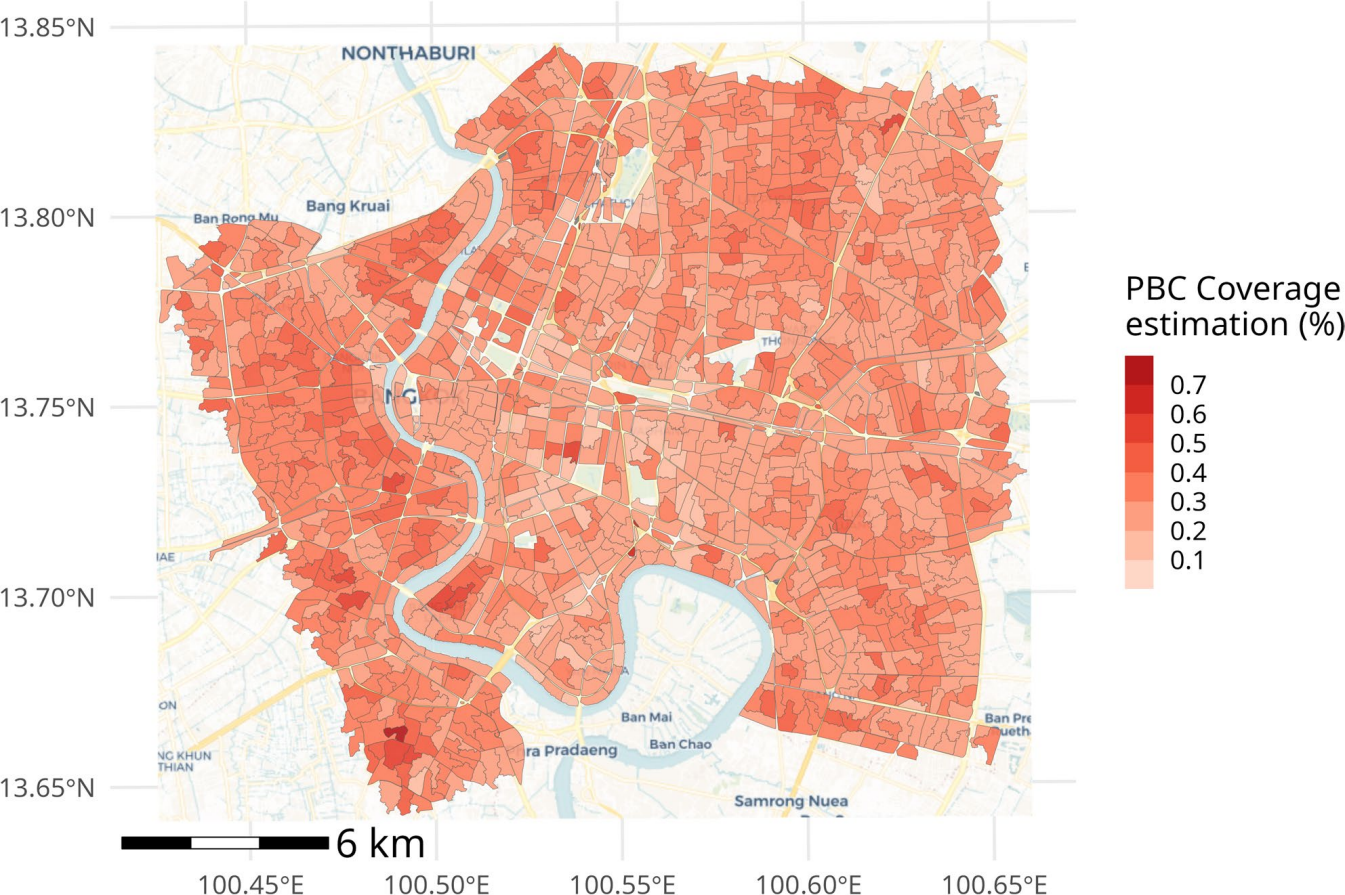
Estimated PBC cluster coverage across central Bangkok. Spatial prediction of PBC cluster coverage (in %) derived from the multivariate linear model integrating building density, urban compactness gradient (|Axis 1|) and internal configuration gradient of vegetation (Axis 3)

For the extrapolation to the whole city, we computed a proxy of built-up linear density for each spatial unit, defined as the number of buildings (Google Open Buildings) divided by the total length of the road network (OpenStreetMap^2^) within the unit, considering only secondary, tertiary, residential and service roads. This ratio captures the intensity of the built fabric along accessible urban interfaces. We then combined this indicator with the PCA coordinates on axes 1 and 3, using the calibrated relationship observed in the sampled zones, to estimate PBC cluster coverage across Bangkok. The resulting spatial distribution reveals a structured yet heterogeneous metropolitan pattern (Fig. 11).

The predicted map of PBC cluster coverage can be interpreted in relation to the two main morphological gradients (Axis 1 and Axis 3; Fig. 1), together with the linear building density proxy used as a predictor of built-up intensity in the model. The spatial pattern does not reproduce a simple center-periphery structure. Although high predicted values are frequent in compact central areas (high positive values on Axis 1), the distribution is modulated by the internal configuration captured by Axis 3. Areas combining strong built density (Axis 1) with larger, more continuous vegetated patches (Axis 3 positive values) display the highest estimated coverage. Conversely, some dense central sectors exhibit only moderate predicted coverage, reflecting less favorable internal organization of interstitial spaces. Similarly, some peripheral areas with moderate compactness but specific structural configurations also show elevated predicted values (south-east part of central Bangkok).

These spatial contrasts suggest that, beyond built intensity alone, the internal organization of interstitial spaces may modulate the local availability and persistence of potential breeding containers. In this study, interstitial organization refers to the fine-grained spatial arrangement of residual micro-spaces generated by the articulation of buildings, parcels, and infrastructures. This organization likely influences micro-environmental conditions such as shading, moisture retention, and object accumulation, which in turn affect the spatial distribution of potential breeding containers.

To assess the coherence of this extrapolation across spatial scales, the zone-level predicted values were compared with the observed route-level PBC cluster coverage in the 36 sampled zones. This comparison should be interpreted as a scale-transfer consistency assessment, rather than as an independent predictive validation.

**Fig. 12:**
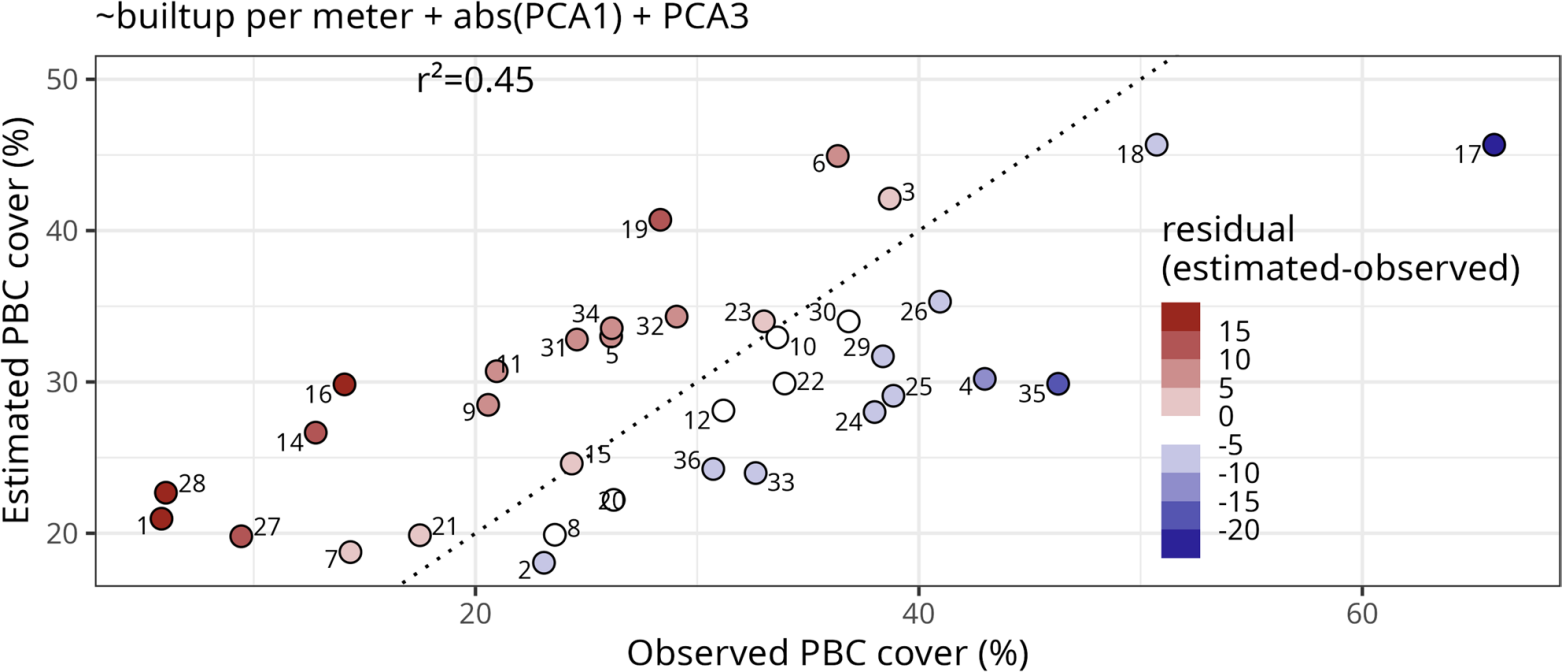
Comparison between observed route-level and predicted zone-level PBC cluster coverage in the 36 surveyed zones. Scatterplot comparing observed PBC cluster coverage (in %) and the corresponding zone-level values predicted by the multivariate linear model including built-up linear density, |PCA Axis 1| and PCA Axis 3. The dotted line represents the 1:1 agreement line. This figure illustrates the coherence of the scale transfer from route-based calibration to zone-level estimation. Point colors indicate residuals, with red tones representing overestimation and blue tones representing underestimations. R² indicates the strength of the correspondence between observed route-level values and zone-level predicted values across the 36 sampled zones

The comparison between observed route-level and predicted zone-level PBC cluster coverage (Fig. 12) suggests that the model captures part of the spatial structure of PBC aggregation when transferred from the surveyed-route scale to the broader zone scale (R² = 0.45). Most points are distributed around the 1:1 line, suggesting that the combined effect of building density and morphological configuration captures the general structure of aggregation patterns across sampled environments. Consequently, the model should be interpreted as providing a structurally informed approximation of PBC cluster coverage rather than a predictive tool in a strict statistical sense.

Model bias can be visualized through the regression residuals. Positive residuals (overestimation, e.g. 1, 28, 27, 14, 16) are more frequent among low-coverage zones belonging to class 1 (Fig. 9), whereas negative residuals (underestimation, e.g. 17, 35, 4, 25, 24 or 33) are more pronounced in several high-coverage sites belonging to class 4 (Fig. 9).

This pattern suggests that extreme aggregation contexts, i.e. zones with very high or very low PBC cover, are not fully accounted for by the structural predictors included in the model. While urban morphology provides a meaningful explanatory baseline, nearly half of the observed variability remains attributable to local-scale factors not explicitly captured by the current specification.

## Discussion

*Urban morphology as a structural driver of breeding container availability*.

This study shows that the spatial availability and aggregation of outdoor potential breeding containers (PBC) in Bangkok are partly structured by urban morphology. Linear built-up density alone explains a substantial share of variability in PBC cluster coverage (R² = 0.41), and the inclusion of PCA-derived descriptors of urban morphology (|Axis 1| and Axis 3) further improves explanatory power (R² = 0.54). These results indicate that breeding-site aggregation is not driven by density alone, but also by the internal organization of interstitial and vegetated spaces within the built fabric. In this perspective, Axis 3 should not be interpreted as a direct predictor of overall container abundance, but rather as a descriptor of the finer spatial organization, clustering, and coverage of potential breeding containers.

**Fig. 13:**
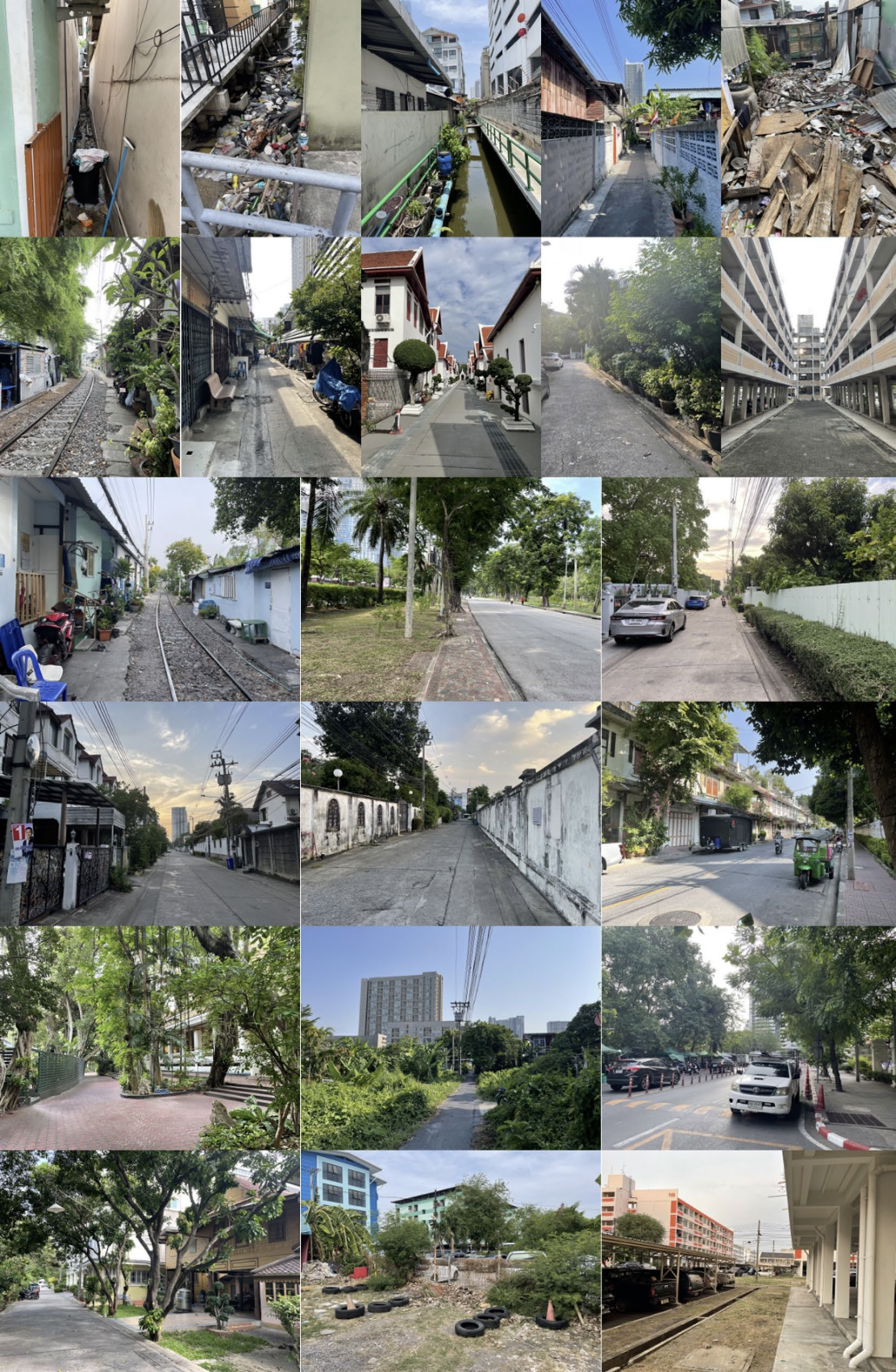
Examples of urban interstitial spaces observed across the study area. The pictures illustrate a diversity of residual and marginal micro-spaces produced by the fragmentation of the built environment, including narrow side alleys between buildings, infrastructure margins (railways, canals, underpasses), rear-lot storage areas, informal dumping areas, vegetated strips along walls or roads, and semi-private residential lanes. These configurations combine shading, limited maintenance, object accumulation, and variable moisture conditions, thereby creating heterogeneous micro-environments potentially favorable to the presence of breeding containers (Photo: E. Daudé, Bangkok, 2024)

In this study, interstitial organization emerges as a key structuring element (Fig. 13). It refers not merely to the proportion of unbuilt space, but to the fine-grained morphological and functional configuration of residual micro-spaces generated by the articulation of buildings, parcels, and infrastructures. These interstices encompass the spaces between built elements and are characterized by their spatial distribution (dispersed or clustered), connectivity (continuous or enclosed), degree of openness, exposure conditions, and modes of appropriation through everyday practices such as storage, vegetation management, or informal deposition. Such configurations influence micro-environmental conditions, including humidity retention, shading, and object accumulation, that directly affect the availability and persistence of potential breeding containers.

The morphological expression of these interstitial spaces varies along the compactness gradient captured by Axis 1 and according to the degree of spatial fragmentation described by Axis 3. In highly compact environments, interstices typically correspond to narrow side alleys, confined gaps between buildings, underpasses, and infrastructure margins. In more open or fragmented configurations, they take the form of rear-lot spaces, vegetated margins, informal storage areas, and transitional zones between private and public domains. Although often limited in surface area, these spaces exhibit distinct intra-zone spatial arrangements, ranging from dispersed containers to tightly aggregated clusters. Thus, beyond built intensity alone, the internal spatial arrangement and functional appropriation of interstitial spaces appear to modulate local vector carrying capacity and help explain why areas with comparable built-up densities may exhibit markedly different PBC coverage.

Field observations illustrate how similar structural outcomes can arise through different mechanisms. Zone 17 (Khlong Toei), representative of dense informal settlement patterns, combines extreme built compactness with constrained waste management and extensive informal storage, resulting in very high PBC densities and strong intra-zone aggregation (180 PBC/km). Conversely, Zone 35 (Chan Kasem) also exhibits high PBC densities (162 PBC/km), but primarily driven by routine domestic practices such as the accumulation of flowerpots along streets. These contrasted cases support the interpretation that urban morphology defines a structural envelope for container accumulation, while local practices determine its realized intensity.

### Non-linear effects of urban compactness

The quadratic relationship between PBC density and the compactness gradient (Axis 1) reveals a U-shaped association: densities peak in both highly compact and highly open urban configurations, whereas intermediate morphologies are associated with lower and more variable values. In compact environments, aggregation may result from limited interstitial space, accumulation dynamics, and intense anthropogenic pressure. In low-density residential settings, higher densities may instead reflect domestic practices such as gardening, outdoor storage, and the presence of numerous small artificial containers. Intermediate configurations may correspond to more regulated or spatially balanced environments, limiting large-scale accumulation processes.

Although the explanatory power of Axis 1 alone remains moderate (R² = 0.17), this non-linear pattern highlights the importance of considering morphological extremes when assessing distribution of water-holding containers. The contrast between zone 4 (Prueksasiri Village), a high-density residential complex (154 PBC/km), and zone 24 (Wat Pathum Wanaram), a temple complex with extensive landscaped areas and numerous containers (136 PBC/km), illustrates how distinct urban configurations can converge toward similar levels of container density through different mechanisms.

From a public health perspective, these results suggest that vector control strategies should not focus exclusively on dense central areas, but also consider specific peripheral configurations that structurally favor container proliferation.

### Limits of structural prediction and the role of local practices

Despite the relatively strong performance of the multivariate model, residual analysis reveals systematic deviations. High-coverage zones tend to be underestimated, while some low-coverage zones are overestimated, and approximately half of the observed variability remains unexplained (R² = 0.45 in internal consistency assessment).

Qualitative field observations (Appendix 1, Figure S2) suggest that local socio-environmental factors likely account for these discrepancies. Informal storage practices, waste accumulation, maintenance regimes, presence of flowerpots, and micro-topographic conditions contribute to aggregation patterns beyond what can be inferred from morphological structure alone [4]. For instance, zone 23 (Wat Daowaduengsaram) shows a high proportion of large containers despite moderate overall density, reflecting local practices rather than structural determinants. This limitation reflects the inherent difficulty of capturing fine-scale socio-environmental practices through structural descriptors alone, and highlights the complementary role of behavioral and local observational data.

Urban morphology thus establishes a structural baseline that conditions the probability of container accumulation, while local socio-environmental practices modulate its intensity. This highlights the importance of integrating behavioral and community-level processes into risk assessment frameworks.

### Implications for entomological surveillance and control

By defining spatial units based on morphological coherence rather than administrative boundaries, the proposed typology provides an operational framework to reduce aggregation bias and improve the ecological interpretability of container-based indicators. In this perspective, the proposed typology can be interpreted as an operational response to the Modifiable Areal Unit Problem (MAUP), by defining spatial units based on morphological and ecological coherence rather than administrative or arbitrary boundaries.

The PAM-derived classification further shows that similar levels of cluster density may correspond to distinct spatial configurations, with implications for control strategies. Diffuse aggregation patterns may require broad community engagement and routine container management, whereas highly clustered environments may benefit from targeted interventions focusing on localized hotspots and specific practices.

The extrapolated map should therefore be interpreted as a spatial prioritization tool identifying structurally favorable urban contexts, rather than as a precise predictor of fine-scale container density.

### Methodological considerations and limitations

Several limitations should be acknowledged. First, the analysis is based on 36 surveyed zones, which constrains statistical power and limits model complexity. Second, the reported R² values correspond to internal model fit rather than independent validation, and predictive performance in unsurveyed areas remains to be assessed.

Third, the proxy used for built-up linear density, based on building counts (Google Open Buildings) and road network length (OpenStreetMap), may introduce measurement uncertainty due to data completeness and classification inconsistencies. The exclusion or limited accessibility of gated communities may also bias the representation of low-density environments. In addition, the restriction of observations to visually accessible areas along surveyed routes may introduce variability in detection depending on local visibility conditions.

Finally, the study focuses on potential breeding containers rather than directly measuring larval productivity or adult mosquito abundance, which limits inference about realized vector populations. The proposed framework should therefore be interpreted as a method for mapping potential distribution of breeding containers rather than actual vector density. Linking these structural patterns to entomological indicators and dengue transmission remains a key direction for future research.

### Toward a spatially explicit early-warning framework

The metropolitan extrapolation reveals a spatial pattern that does not simply reproduce a center-periphery gradient, but instead reflects the interaction between built intensity and internal spatial configuration. Areas combining high built density with favorable interstitial organization promoting object accumulation, shading, and moisture retention exhibit the highest predicted PBC coverage.

These findings suggest that urban morphological descriptors may serve as stable baseline predictors for identifying structurally favorable environments for vector habitat formation. The increasing availability of geospatial data (e.g., satellite imagery and large building databases) makes it possible to characterize urban landscapes across a wide range of contexts [35, 40, 41]. When combined with climatic, socio-economic, and entomological data, such descriptors could contribute to spatially explicit early-warning systems for dengue transmission risk.

Ultimately, the study supports a multiscale perspective in which urban morphology defines the structural envelope of water-holding containers potential, while local practices determine its realized intensity. Bridging these scales remains a central challenge for predictive urban epidemiology.

## Conclusion

This study shows that the spatial availability and aggregation of outdoor potential breeding containers in Bangkok are partially predictable from urban landscape structure. Building density provides a first-order explanation, but multivariate descriptors capturing compactness intensity and internal configuration of built and vegetated spaces substantially improve model performance. Importantly, field observations indicate that local socio-environmental practices modulate these structural effects, explaining why extreme aggregation contexts are not fully captured by morphology alone [40].

By framing the city as a mosaic of structurally favorable environments for the accumulation of water-holding containers, the proposed typology offers an operational basis for entomological surveillance and spatial targeting of vector control [36]. Rather than predicting realized mosquito abundance directly, it identifies the urban structural conditions under which breeding-site accumulation is more likely to occur. Future work should therefore connect these container-based patterns to mosquito productivity and dengue transmission indicators.

## Supplementary Information

Supplementary Material 1

## Data Availability

The curated dataset supporting the findings of this study is publicly available in the following open-access repository: Daudé, E., Cebeillac, A. (2026). Data on Potential Mosquito Breeding Containers in Bangkok (Thailand), 2023-2024 [Data set]. Zenodo. https://doi.org/10.5281/zenodo.18698866.

## Footnotes

1
https://sites.research.google/gr/open-buildings/.

2
https://www.openstreetmap.org/.
