# Supplementary material for: Mapping urban Aedes aegypti breeding containers: landscape-driven analysis in Bangkok, Thailand
Source: Int J Health Geogr. 2026 May 13;25:40. doi: 10.1186/s12942-026-00469-3 (PMC13397698; doi:10.1186/s12942-026-00469-3)
Supplement: Supplementary file 1 — Supplementary Material 1 [file 12942_2026_469_MOESM1_ESM.pdf]

Appendix 1.

Description of the surveyed zones and distribution of potential containers by volume class in Bangkok, Thailand.

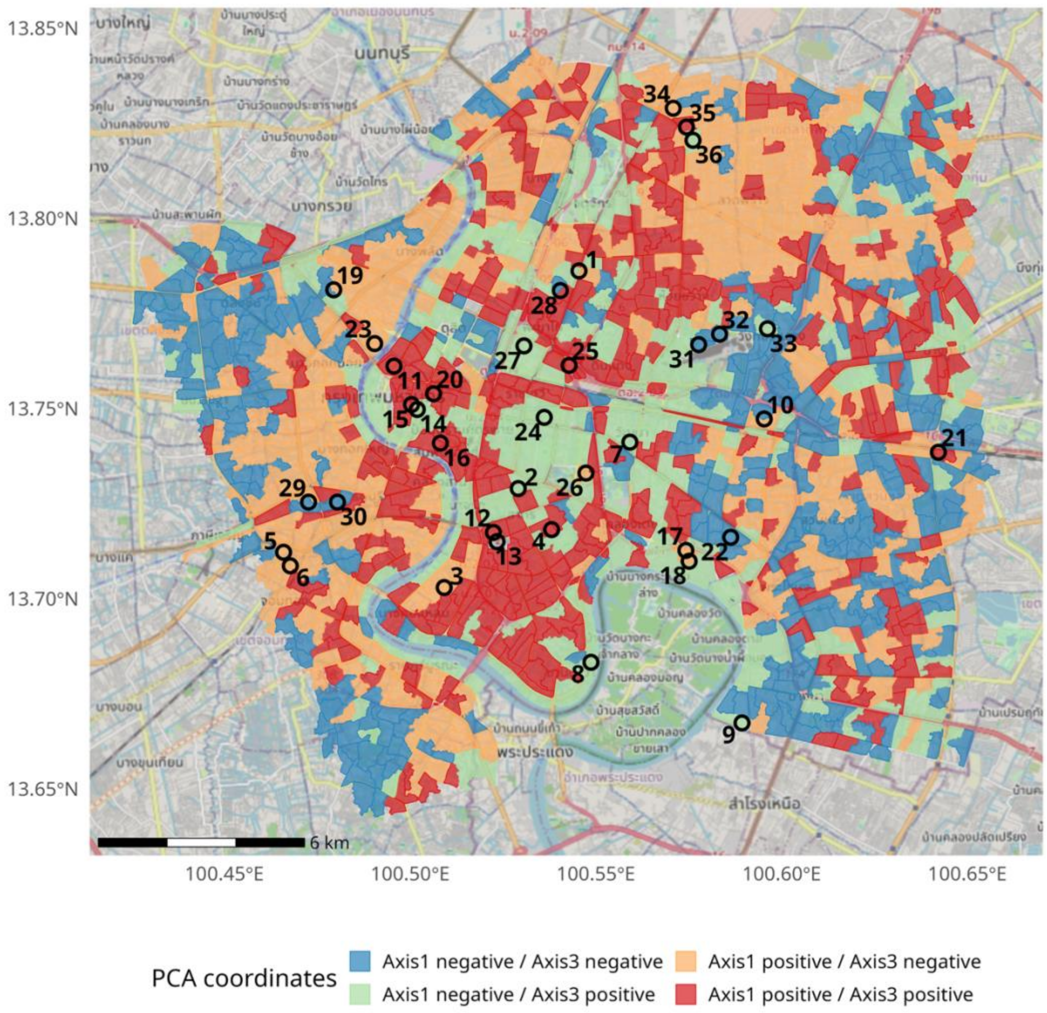

Figure S1. Spatial distribution of the 36 surveyed zones across the Bangkok study area according to the sampling design. The map shows the location of the 36 field-surveyed zones used for potential breeding container (PBC) data collection across the Bangkok study area. Background colors represent the four broad typological strata defined by the sign of the PCA coordinates on Axis 1 (built-up density and urban compactness gradient) and Axis 3 (internal configuration of built and vegetated spaces). This figure illustrates both the spatial coverage of the field survey and the representativeness of the sampled zones with respect to the diversity of urban landscape configurations considered in the study. Zone IDs correspond to those used throughout the manuscript.

Figure S2: Description of the study zones and distribution of potential breeding containers (PBCs) by volume class. *For each surveyed zone (ID), the distribution of PBCs across five container volume classes is represented using a square of identical size corresponding to 100% of the observed containers. The colored portion of each square indicate the relative proportion of PBCs in each class : blue (< 0.5 L), green (0.5–1 L), yellow (1–2 L), orange (2–5 L) and red (> 5 L), while the numbers indicate the corresponding absolute counts. The map of each zone includes the survey route (red lines) and the recorded potential breeding containers (PBCs, grey dots).*

Data available at : [*https://analytics.huma-num.fr/UMR_IDEES/MO3/zones_leafl.html*](https://analytics.huma-num.fr/UMR_IDEES/MO3/zones_leafl.html)

**ID**	**Zone description**
**1**	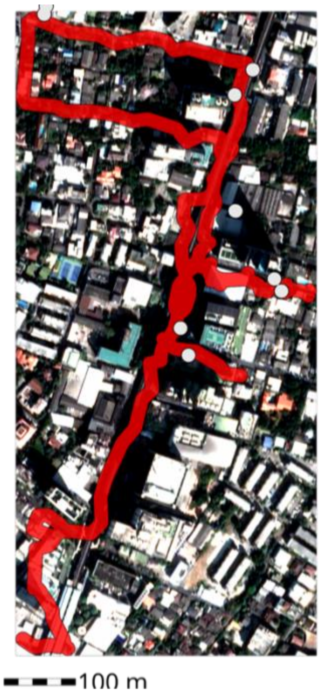 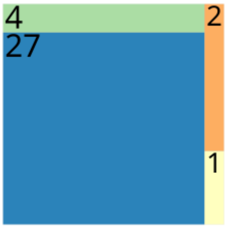 **Ari–Phaya Thai** is an affluent neighborhood characterized by a mix of high-rise residential buildings (approximately 10–15 storeys) and individual houses with private gardens and interior courtyards. The urban fabric combines vertical residential density with enclosed green and semi-private outdoor spaces.<br><br>*Average number of PBC/km:* 4

**2**	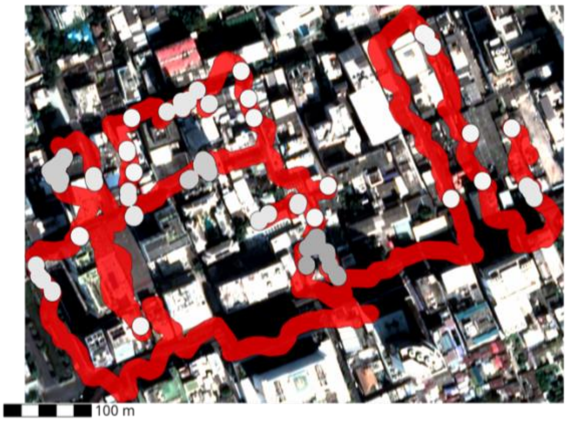<br><br>**Silom** is a central tourist district characterized by high-rise buildings and streets densely occupied by tourism-oriented activities (bars, restaurants, massage parlors). A high number of potential breeding containers (PBCs), predominantly located beneath flowerpots, were observed, mainly around outdoor terraces associated with bars and restaurants.<br>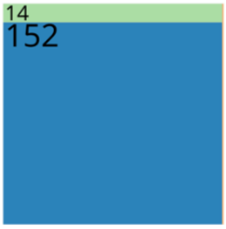<br><br>*Average number of PBC/km: 47*
**3**	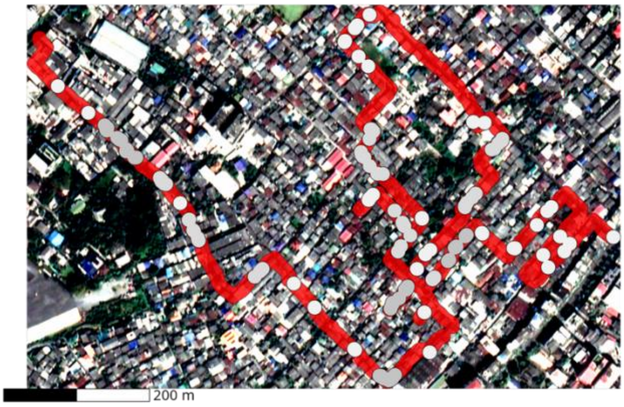<br><br>**Pratanpon** is a lower-income residential neighborhood characterized by small wooden houses, often with corrugated metal (tin) roofs. The built environment is associated with a high density of potential breeding containers, including numerous flowerpots and unmanaged waste materials that may serve as mosquito breeding sites.<br>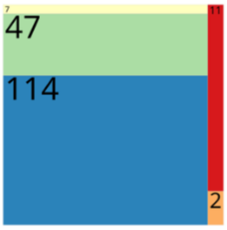<br><br>*Average number of PBC/km: 66*

**4**	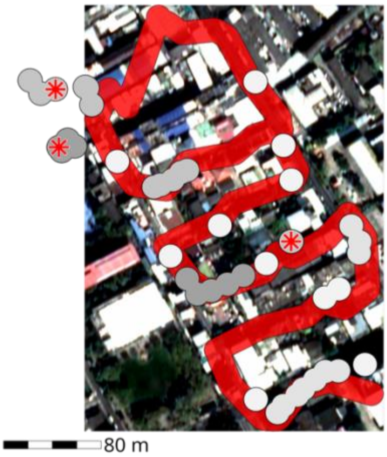 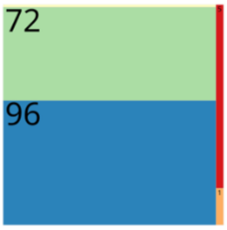 **Prueksasiri Village** is a residential neighborhood characterized by mid-rise apartment buildings (approximately 5–7 storeys). The internal alley network is narrow, with a high presence of flowerpot saucers observed along circulation spaces, constituting numerous potential breeding containers.<br>*Average number of PBC/km: 154*
**5**	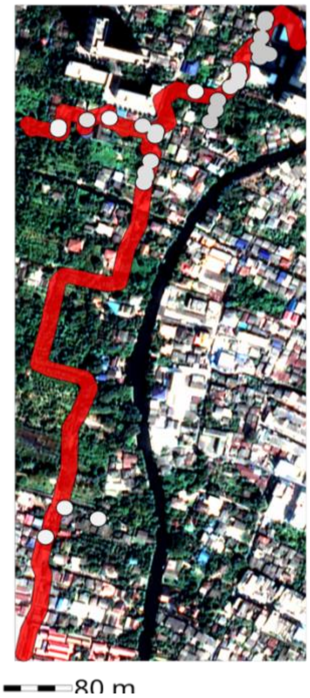 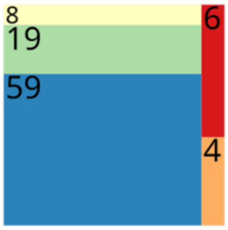 **Wutthakat** is a sparsely built, highly vegetated area characterized by a limited number of residential houses, many of which are in poor physical condition. The surrounding gardens are largely unmanaged, with dense vegetation and neglected outdoor spaces.<br>*Average number of PBC/km: 61*
**6**	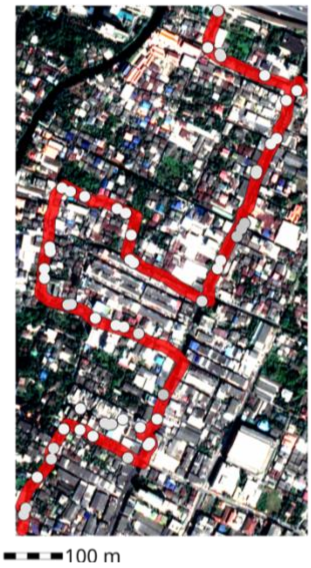 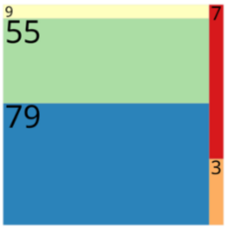 **Wutthakat-bis** is a socio-economically underprivileged neighborhood characterized by old, partly dilapidated low-rise buildings (2–3 storeys), primarily aligned along main roads. Numerous abandoned objects were observed in secondary and back alleys, constituting a high density of potential breeding containers.<br>*Average number of PBC/km: 80*

**7**	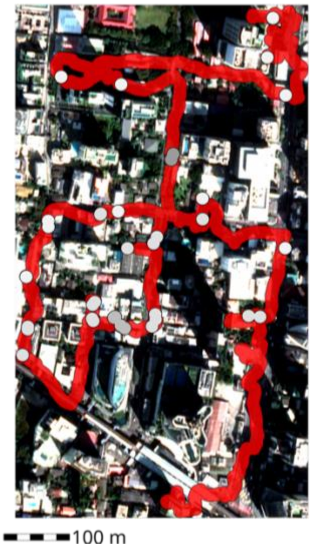 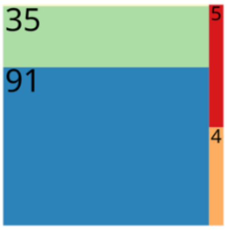 **Sukhumvit** is a highly touristic and intensely frequented district characterized by very high-rise buildings and a dense concentration of hotels, many of which include landscaped gardens or park-like areas. The area is marked by high levels of activity and noise, reflecting its strong commercial and tourism functions.<br>*Average number of PBC/km: 41*
**8**	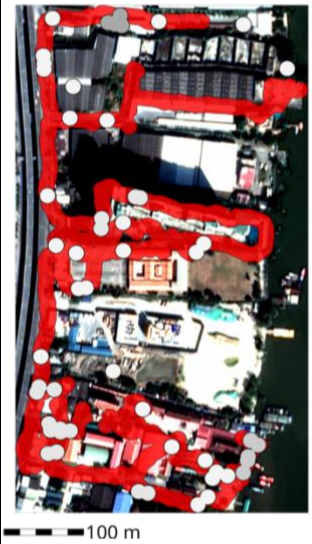 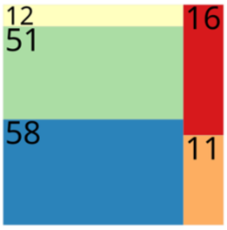 **Phanrawi 2 Village** is a mixed-use area combining residential housing with large port-related warehouse facilities. The road infrastructure serving these warehouses creates wide interstitial spaces, including extensive parking lots and numerous vacant or unused areas between roadway structures.<br>*Average number of PBC/km: 43*
**9**	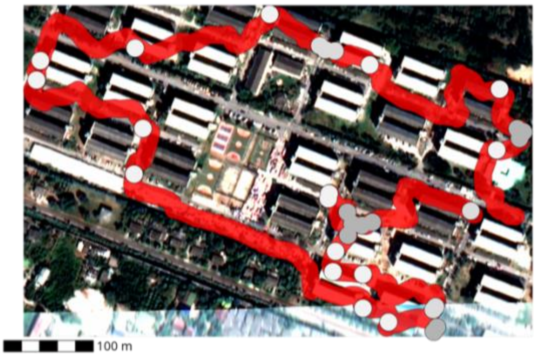 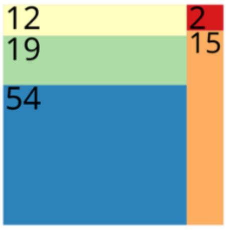 **Bang Na** is a lower- to middle-income residential neighborhood characterized by residential buildings separated by parking lots and organized around central concrete courtyards. The area is generally well maintained. Despite this, a high number of potential breeding containers (PBCs) were observed throughout the courtyards and surrounding spaces.<br>*Average number of PBC/km: 50*

<table>
<tr>
<td>10</td>
<td>
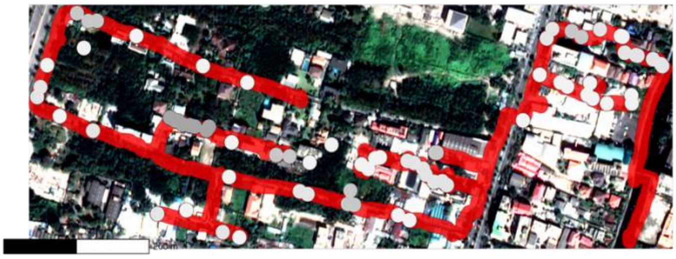

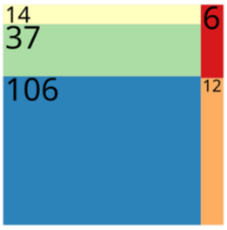

Bang Kapi is a low-density residential neighborhood characterized by a heterogeneous built environment, combining luxury houses with smaller, more modest buildings. Numerous vacant lots are present, many of which are partially occupied by accumulated waste and debris.

Average number of PBC/km: 90
</td>
</tr>
<tr>
<td>11</td>
<td>
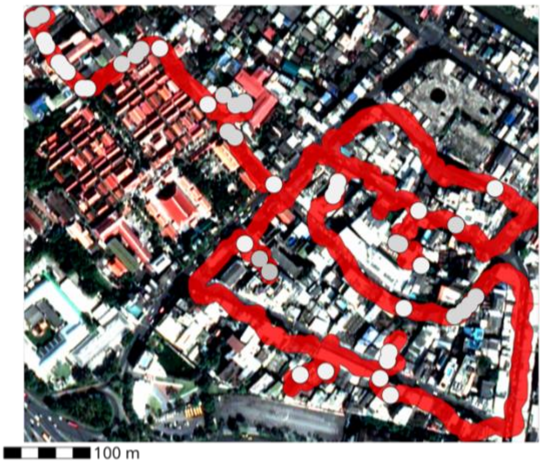

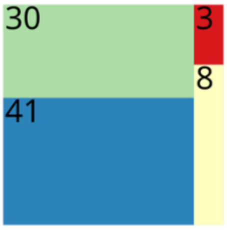

Khaosan Road is a major tourist area located north of Phra Nakhon, characterized by wide streets densely occupied by bars, restaurants, and tourist stalls. The area includes numerous hotels spanning a wide range of standards, from modest accommodations to larger establishments with gardens and swimming pools.

Average number of PBC/km: 38
</td>
</tr>
<tr>
<td>12</td>
<td>
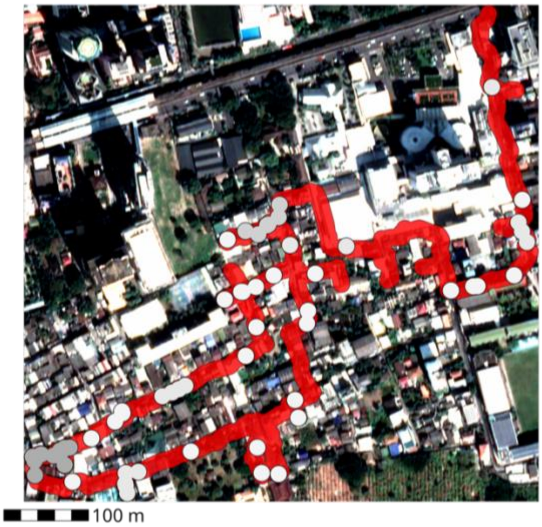

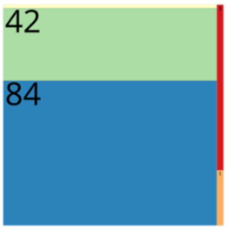

Sathon is an old, middle - to high income residential neighborhood characterized primarily by courtyard houses. The urban fabric includes a limited number of abandoned or vacant plots, some of which contain accumulated waste and debris.

Average number of PBC/km: 60
</td>
</tr>
</table>

<table>
<tr><td>13</td><td>

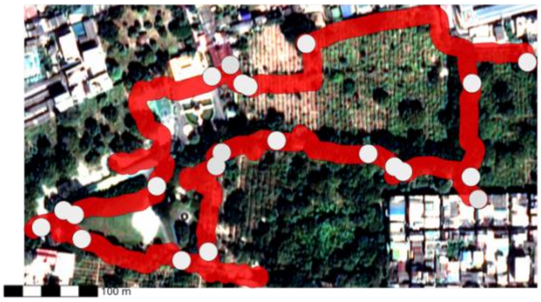

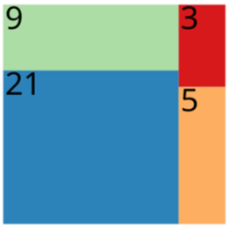

Park of Rim Sathon Village is a central open space located within an old residential neighborhood, encompassing a Chinese cemetery, outdoor fitness areas, and sports fields. Despite dense and invasive vegetation between the tombs and the presence of a few water-holding saucers, little to no litter was observed. The site is regularly frequented by local residents for jogging and outdoor exercise.

Average number of PBC/km: 27

</td></tr>
<tr><td>14</td><td>

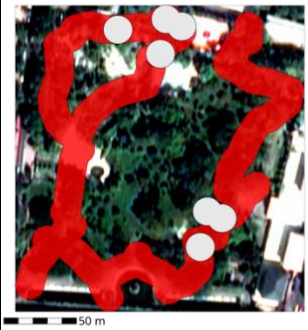

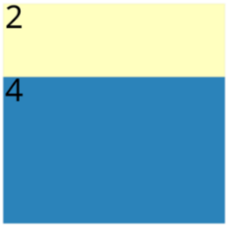

Rommaninat Park is a small, well-maintained urban park equipped with outdoor weight-training facilities and basketball courts.

Average number of PBC/km: 12

</td></tr>
<tr><td>15</td><td>

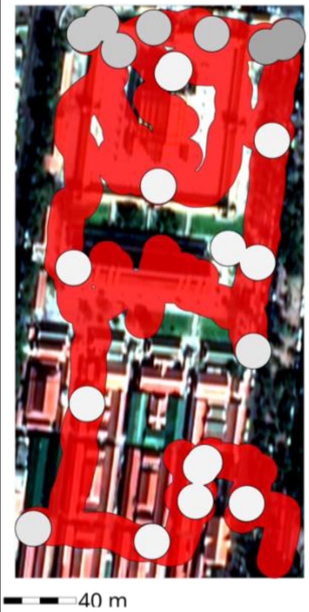

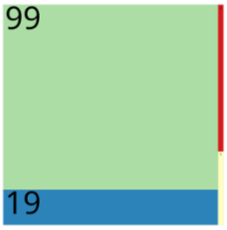

Wat Suthat Thepwararam Ratchaworahawihan is a well-maintained Buddhist temple complex characterized by renovated temple buildings and monks' residential quarters. Very few potential breeding containers were observed across the site, with the notable exception of a continuous line of flowerpots with saucers located along the northern roadside.

Average number of PBC/km: 87

</td></tr>
</table>

**16**	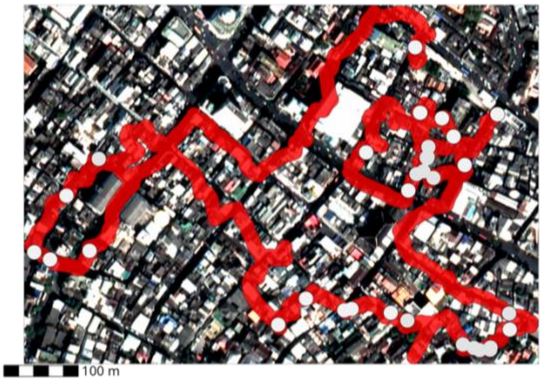 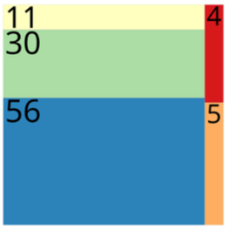 **Chinatown / Yaowarat** is a very dense and highly active urban district characterized by intense commercial activity along both main roads and a dense network of narrow alleyways. Numerous street stalls occupy these circulation spaces, many of which are partially sheltered from rainfall by temporary curtains or tarpaulins. The area contains large numbers of stored or piled objects and very limited vegetation.<br><br>*Average number of PBC/km: 20*
**17**	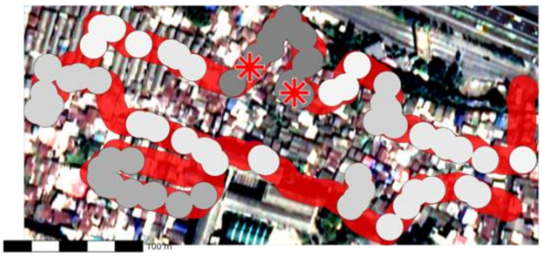 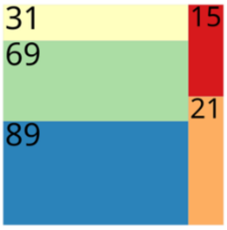 **Khlong Toei** is a low-income residential neighborhood (a slum) characterized by small houses aligned along narrow streets, predominantly constructed from wood and sheet metal. Outdoor spaces are densely cluttered with flowerpots, tubs, and containers, and substantial accumulations of unmanaged waste are present between and beneath houses.<br><br>*Average number of PBC/km: 180*
**18**	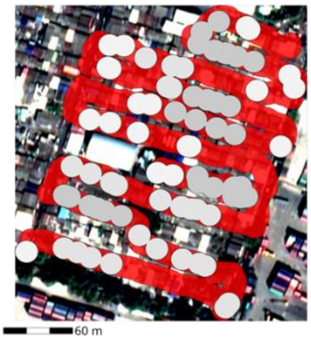 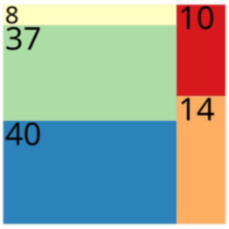 **Khlong Toei (southern sector)** is located south of the previous site, characterized by a mixed housing stock combining brick-and-cement constructions with wooden and sheet-metal dwellings. Alleyways are less crowded, with fewer obstructions. Potential breeding containers are present but less numerous, mainly consisting of flowerpots with saucers and a limited number of large water-holding containers.<br><br>*Average number of PBC/km: 100*

<table>
<tr><td>19</td><td>
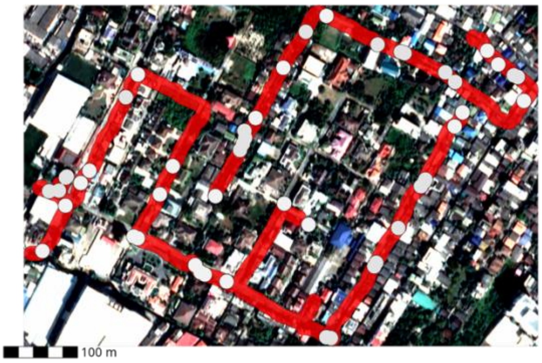

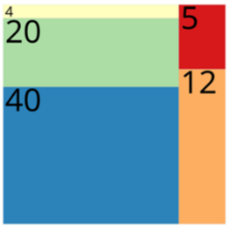

Bang Phlat is an affluent residential neighborhood close to pink lao mall and composed primarily of large single-family homes set within private gardens and park-like environments. Many residences exceed 300 m² and are surrounded by extensive yards. A proportion of the housing stock has been unoccupied for extended periods (several months to years), resulting in partially unmanaged residential plots.

Average number of PBC/km: 51
</td></tr>
<tr><td>20</td><td>
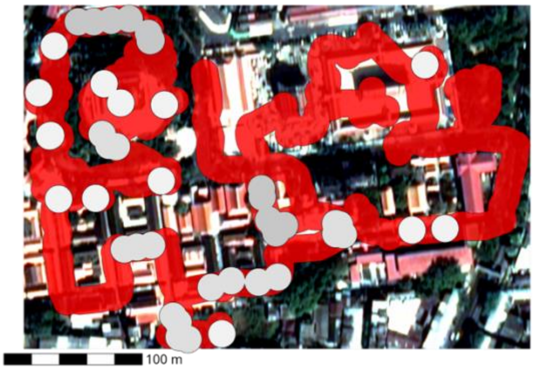

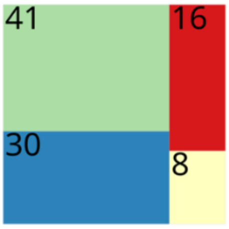

Wat Saket is a major religious site in Bangkok, situated on an elevated mound and characterized by high maintenance standards. Potential breeding containers are generally limited and spatially concentrated around monks’ living quarters, with additional large water jars used for watering purposes.

Average number of PBC/km: 47
</td></tr>
<tr><td>21</td><td>
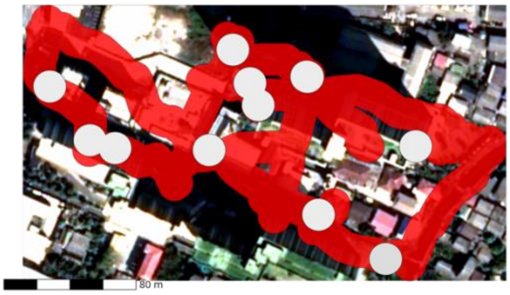

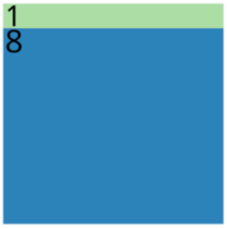

Hua Mak is a residential neighborhood characterized by very large high-rise apartment buildings located in close proximity to metro and BTS lines. At the base of these buildings, smaller houses with private gardens are present. The area is generally very well maintained, with very few visible potential breeding containers.

Average number of PBC/km: 18
</td></tr>
</table>

**22**	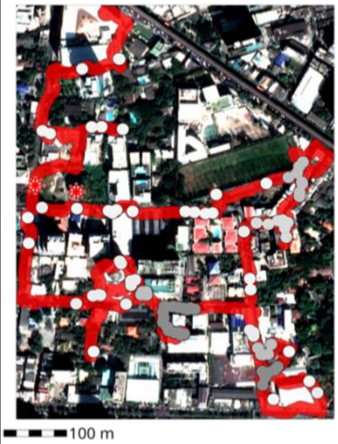 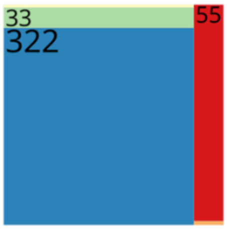 **Ekkamai - Sukuhmvit 42** is an upper-income residential area with extensive vegetated spaces between mid-rise (5–10 storeys) and occasional high-rise buildings. These open spaces may provide conditions conducive to the presence of potential breeding containers.<br>*Average number of PBC/km: 125*
**23**	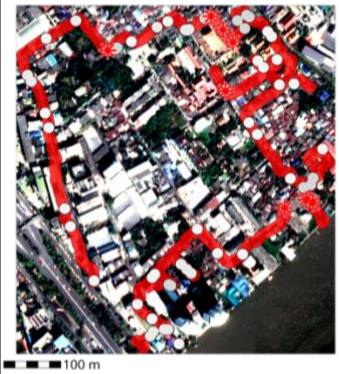 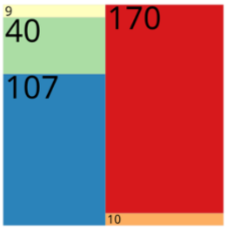 **Wat Daowaduengsaram** is a riverside area organized around a temple, combining a compact canal-side urban village of small houses with residential buildings located along a major highway.<br>*Average number of PBC/km: 121*
**24**	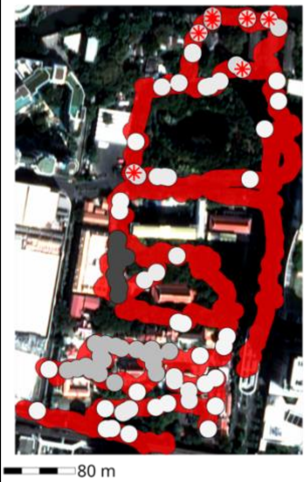 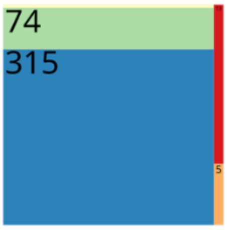 **Wat Pathum Wanaram** is a temple complex situated between two major commercial centers in Bangkok. The site includes religious buildings, residential facilities for monks and students, a school, and a northern park area. Extensive gardens and tree cover are present, with numerous flowerpots and vases distributed throughout the landscaped spaces.<br>*Average number of PBC/km: 136*
**25**	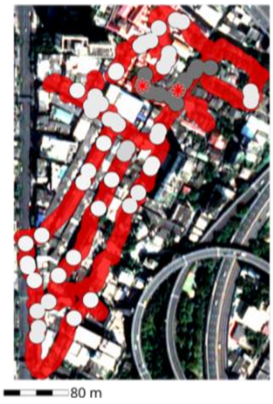 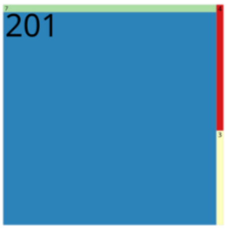 **Soi Ratchaprarop and Wat Thatsanarun Sunthrikaram** is a middle-class neighborhood composed of small courtyard houses and low-rise buildings (2–3 storeys) along narrow alleys with numerous flowerpots. The temple occupies the north-western sector, and the area is bounded by a highway to the east.<br>*Average number of PBC/km: 105*

**26**	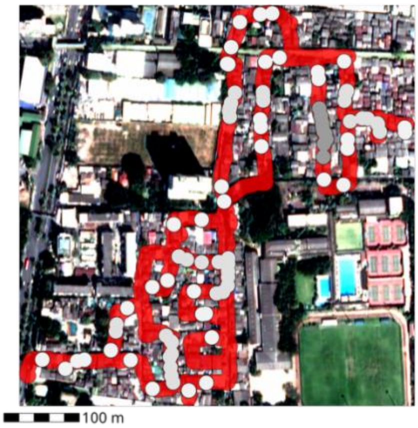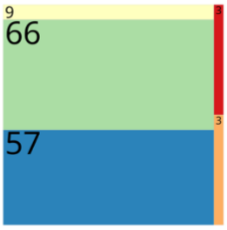<br>**Sanam Khli** is a residential neighborhood located east of Lumphini Park. The area is composed mainly of 1–2 storey single-family houses with concrete inner courtyards and perimeter walls that shield private spaces from the street. Numerous small local food shops are distributed throughout the neighborhood. Vegetation is scarce, limited to a few shrubs, with the exception of a polo field located on the eastern edge of the area.<br>*Average number of PBC/km: 50*
**27**	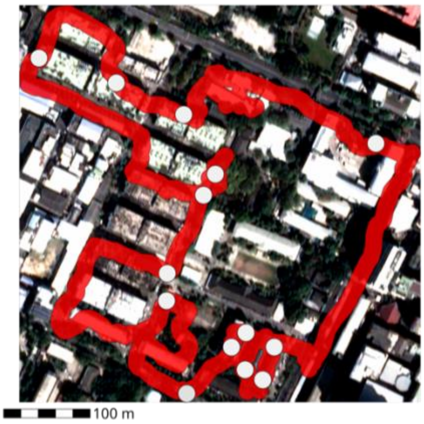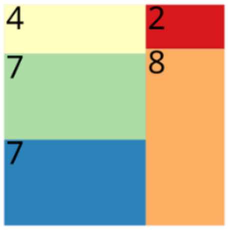<br>**Victory Monument–Ramathibodi** sector is a residential neighborhood characterized by large collective residential buildings of approximately ten storeys, separated by narrow one-lane streets and well-maintained, paved open spaces primarily used for parking. Commercial activity is limited, and the housing stock largely serves employees of the surrounding hospitals and universities.<br>*Average number of PBC/km: 11*
**28**	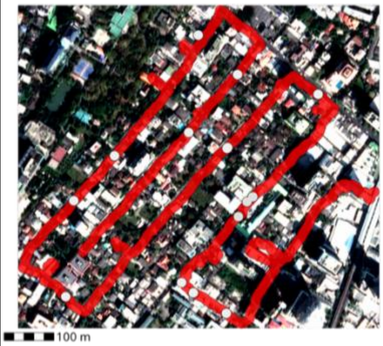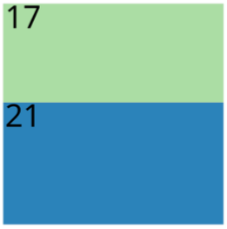<br>**Ari (Soi 1 to 5)** is a high-income residential area characterized by detached houses with gardens and courtyards, alongside well-maintained 4–5 storeys apartment buildings, all enclosed by boundary walls. The predominance of private, enclosed spaces restricts access to much of the environmental setting. The careful maintenance of publicly accessible areas results in a very low observed density of PBCs.<br>*Average number of PBC/km: 10*

**29**	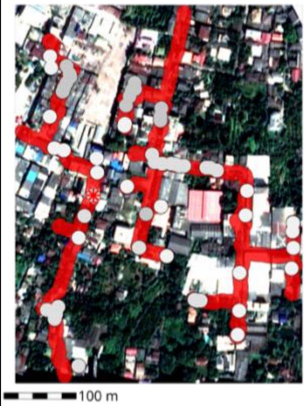<br><br>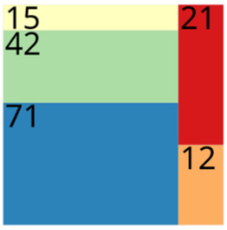<br><br>**Tha Phra – Phet Kasem** is a middle-income residential neighborhood located on the left bank of the Chao Phraya River. The area is composed mainly of small low-rise buildings (2–3 storeys), with occasional ground-floor commercial activities or home-based shops secured by simple metal grilles. Numerous plants and stored objects are present in front of entrances, contributing to a dense and heterogeneous streetscape.<br>*Average number of PBC/km: 84*
**30**	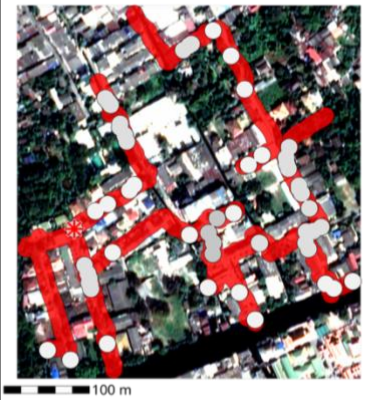<br><br>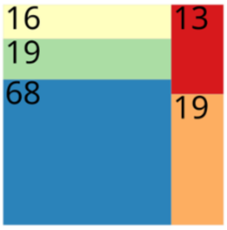<br><br>**Tha Phra East** is comparable to Zone 29, with a slightly more affluent residential profile and a higher level of vegetation due to the presence of interconnected micro-parks. These vegetated open spaces, occasionally used for informal dumping, host the majority of observed potential breeding containers (PBCs), including discarded tires and gardening equipment.<br>*Average number of PBC/km: 70*
**31**	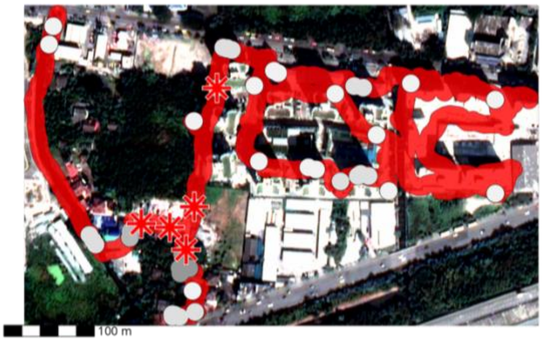<br><br>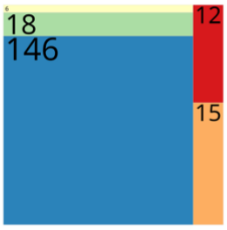<br><br>**Huai Khwang** is a modern, high-income residential area composed of a large complex of approximately twenty similar high-rise buildings (around 20 storeys each). The complex is surrounded by landscaped green spaces and includes numerous flowered areas within its internal courtyards. Several potential breeding containers were observed, particularly in storage areas used for maintenance and gardening equipment.<br>*Average number of PBC/km: 82*

**32**	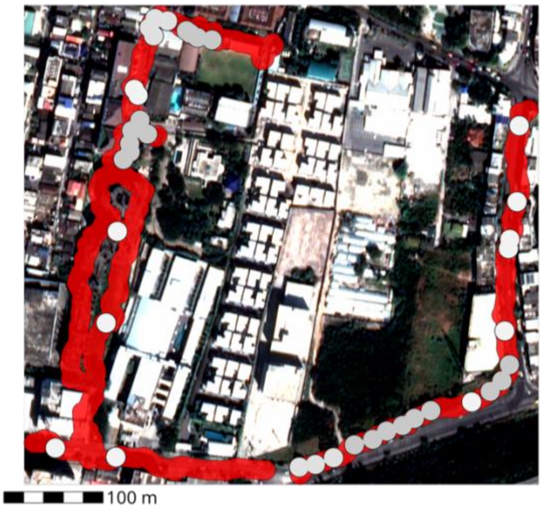 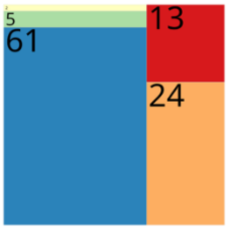 **Huai Khwang East** presents a transitional urban configuration, combining high-rise residential towers (≈20 storeys), mid-rise buildings (≈10 storeys), vacant plots, and ongoing construction sites. The neighborhood was in a phase of active redevelopment during fieldwork.<br><br>*Average number of PBC/km: 56*
**33**	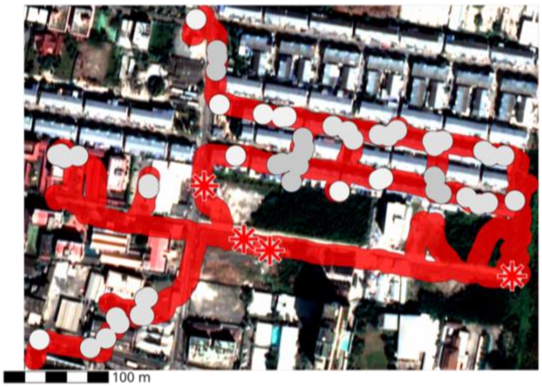 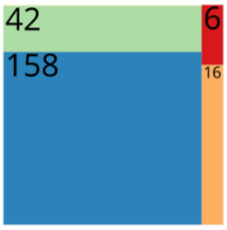 **Wang Thonglang** is a modern residential neighborhood characterized by large terraced houses of 3–4 storeys. Ground floors are commonly used as garages or storage spaces. Streets are well maintained and extensively lined with flowerpots along building fronts.<br>*Average number of PBC/km: 96*
**34**	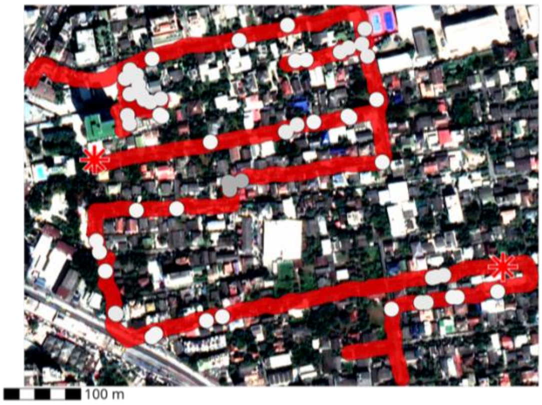 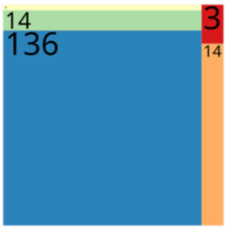 **Chan Kasem**, in the vicinity of BTS Ratchayothin station, is a predominantly residential area characterized by detached, enclosed houses with private gardens. Terraced housing and small-scale food retail activities are present along certain streets. The neighborhood exhibits a high proportion of vegetation, forming a mosaic structure with the built fabric.<br>*Average number of PBC/km: 60*

**35**	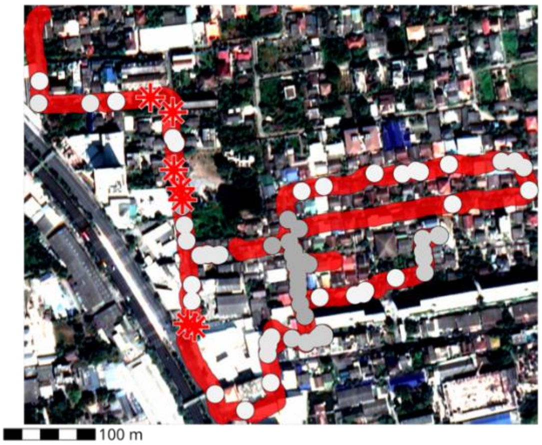<br><br>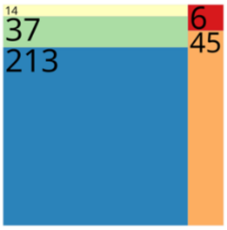<br><br>**Chan Kasem** (southern sector, Zone 34) presents a configuration similar to the adjacent area, though characterized by smaller residential units. Detached houses remain predominant, but with more modest dimensions. A higher density of flowerpots was observed along the streets, contributing to an increased presence of small potential breeding containers.<br>*Average number of PBC/km: 162*
**36**	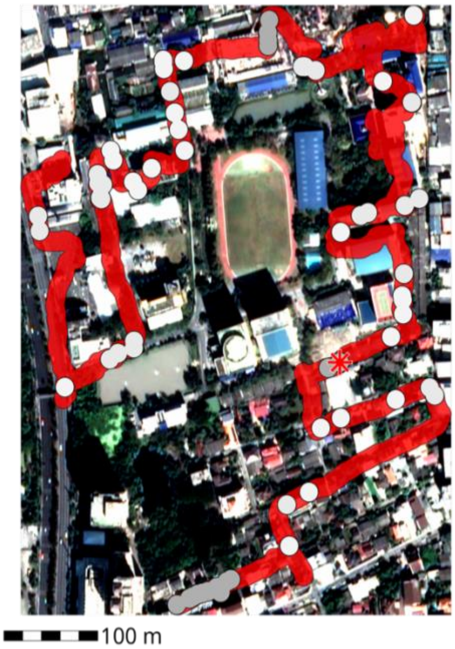<br><br>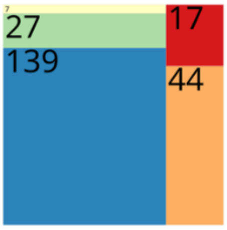<br><br>**Chan Kasem (around Chandrakasem Rajabhat University)** combines mid-rise residential buildings (4–5 storeys) with local retail and service activities linked to the nearby university. The neighborhood includes dispersed vegetated elements, such as small private gardens, shrubs, and sidewalk plantings, either directly in the soil or in containers.<br><br>*Average number of PBC/km: 87*
